# Risk of mortality and complications in people with depressive disorder and co-occurring diabetes mellitus: a systematic review and meta-analysis

**DOI:** 10.1017/S2045796026100882

**Published:** 2026-09-04

**Authors:** Matthew Tsz Ho Ho, Kwun Nam Chan, Will Chi Yuen Chiu, Lucy Lo Wah Tsang, Heidi Ka Ying Lo, Steven Yiu Long Wong, Kenneth Shut Wah Chan, Mimi Mei Cheung Wong, Ho Hon Wong, Wai Chung Lam, Pui Fai Pang, Wing Chung Chang

**Affiliations:** 1 Department of Psychiatry, University of Hong Kong Li Ka Shing Faculty of Medicine, Hong Kong; 2 Department of Psychiatry, United Christian Hospital, Hong Kong; 3 Department of Psychiatry, The University of Hong Kong, Hong Kong; 4 Department of Psychiatry, Queen Mary Hospital, Hong Kong

**Keywords:** depression, diabetes mellitus, epidemiology, health outcomes, meta-analysis, systematic reviews

## Abstract

**Aims:**

People with depressive disorder have increased premature mortality and higher rates of diabetes mellitus than general population. Evidence shows that diabetes may further increase their risk of premature death from diabetes-related complications, especially cardiovascular diseases (CVDs). Earlier studies examining depression-associated outcomes in diabetes patients have shown mixed results and were hindered by important limitations, especially the use of self-reported questionnaires to ascertain depression, causing misclassification bias by identifying subclinical symptoms or diabetes distress. Associations of depression with specific diabetes complications have not been systematically evaluated. This meta-analysis aimed to investigate the risk of mortality and complications among patients with depression and co-occurring diabetes (depression-diabetes group) relative to patients with diabetes-only (diabetes-only group), on their all-cause mortality rates, and if applicable cause-specific mortality rates, and occurrence of specific diabetes complications.

**Methods:**

We systematically reviewed and quantitatively synthesized diabetes-related outcomes in patients with depression by searching Embase, MEDLINE, PsycInfo and Web-of-Science from inception to 20 December 2024, and included studies that examined mortality and complication outcomes in depression-diabetes group relative to diabetes-only group. Results were synthesized by random-effects meta-analytic models, with stratified-analyses (subgroup analyses and meta-regression) by study-level characteristics, including age, gender, study period, geographic region, follow-up duration and nature of diabetes sample. The study was registered with PROSPERO (CRD42024595145).

**Results:**

Twenty-six studies were identified from nine geographic regions. Regarding mortality risk, depression-diabetes group exhibited increased risks of all-cause mortality (RR = 1.30 [95% CI: 1.21–1.39]) and CVD-specific mortality (1.15 [1.02–1.29]) relative to diabetes-only group. Regarding complication risk, depression-diabetes group showed increased risk of complications (1.28 [1.18–1.40]) relative to diabetes-only group, especially in incident-diabetes sample signifying advanced disease stage upon presentation, with stratified-analyses showing higher risk of metabolic complications (1.63 [1.33–1.99]) and cardiovascular complications (1.20 [1.11–1.29]), and lower likelihood of retinopathy (0.84 [0.76–0.94]), albeit comparable rates of cerebrovascular complications (1.36 [0.99–1.87]), nephropathy (1.09 [0.93–1.27]) and peripheral-vascular complications (0.97 [0.79–1.18]). Both overall mortality and complication risks were present in various regions and persisted over time. Heterogeneities were noted and could not be entirely explained by stratified analyses.

**Conclusions:**

Our study demonstrated that patients with depression and co-occurring diabetes were associated with elevated overall mortality risk and complication risk (particularly metabolic and cardiovascular-complications) than non-depressed counterparts, suggesting an overall poorer glycemic control that might eventually drive their earlier death. Comprehensive and multipronged interventions are needed for individualized risk estimation of diabetes-related outcomes, with consequent early interventions to minimize the avoidable physical morbidity and premature mortality in this vulnerable population.

## Introduction

Depressive disorder affects approximately 280 million people worldwide (GBD Mental Disorders Collaborators, 2022) and shortens their lifespan by about 13 years relative to people without the disorder (Chan *et al.*, 2023). Evidence has suggested that physical illnesses could explain about two-thirds of premature mortality in patients with mental disorders (Walker *et al.*, 2015), and recent evidence has further shown that a substantial proportion of mortality in people with depression was due to cardiovascular diseases (CVDs) (Correll *et al.*, 2017; Lo *et al.*, 2025). Type 2 diabetes mellitus is an important CVD risk factor (Vancampfort *et al.*, 2015a) and, in itself, a leading global disability (GBD 2019 Diseases and Injuries Collaborators, 2020), affecting approximately 529 million people (GBD 2021 Diabetes Collaborators, 2023). Diabetes and its complications affect multiple systems, particularly the cardiovascular system, including coronary heart disease and stroke, which are respectively the second and third leading causes of global disability (GBD 2019 Diseases and Injuries Collaborators, 2020).

Existing studies have in fact indicated bidirectional relationships between depression and type 2 diabetes (Fanelli *et al.*, 2025; Pouwer *et al.*, 2026). Accumulating evidence has shown that patients with depression are at increased risk of hyperglycemia and type 2 diabetes compared to those without depression (Vancampfort *et al.*, 2014, 2015b; Lindekilde *et al.*, 2022). Some studies have also demonstrated elevated cytokine and chemokine alterations in depression compared to the comparison (Köhler *et al.*, 2017), implying shared immunological pathways across both depression and diabetes (Moulton *et al.*, 2015). Conversely, diabetes may increase the risk of depression due to factors such as physiological stress and emotional responses to long-term complications (Gonzalez *et al.*, 2011). In addition, the co-occurrence of depression and diabetes is presumably driven by numerous shared risk factors, including unhealthy lifestyle (Pearce *et al.*, 2022) and polypharmacotherapy (Jeffery *et al.*, 2023). As diabetes is a major risk factor for CVD-related death in depression, it is clinically important to confirm the impact of depression on the outcomes of diabetes (Dragioti *et al.*, 2023). A handful of studies have systematically evaluated the associations between depression and diabetes-related outcomes, including mortality and complication risks (van Dooren *et al.*, 2013; Farooqi *et al.*, 2019; Nouwen *et al.*, 2019), but the findings were hampered by several important limitations. First, the use of self-reported questionnaires to define depression in the vast majority of the included studies may identify depressive symptoms at a subclinical level rather than a diagnosis of clinical depression (Roy *et al.*, 2012). Second, these depressive symptoms might indicate diabetes distress (Fisher *et al.*, 2007), which is clinically distinct from depressive disorder, and thus might potentially blur the effect of depressive disorder on outcome estimates. Third, using these tools with variable cut-off values to define depression might lead to an inconsistent assessment of depression across studies, leading to problems with the comparability of depression exposure. Importantly, studies examining the association between depression and diabetes complications were inconsistent, with some showing higher risks (Yoo *et al.*, 2023; Scheuer *et al.*, 2024), and others lower or comparable risks (Wu *et al.*, 2020; Ho *et al.*, 2025a). As the associations of depression with a wide spectrum of specific diabetes complications have not yet been systematically evaluated, quantitative synthesis is indicated to aggregate the results to inform healthcare policy-making, particularly the formulation of tailored preventive approaches of diabetes in patients with depression to improve their clinical outcomes.

To this end, we conducted a systematic review and meta-analysis to examine the risk of mortality and complications among patients with depression and co-occurring diabetes (i.e., depression-diabetes group) relative to patients with diabetes only (i.e., diabetes-only group), with an aim to compare between the two patient groups on their all-cause mortality rates, and if applicable, other cause-specific mortality rates, as well as the occurrence of specific diabetes complications. Additionally, stratified analyses by pre-specified study-level characteristics, including age, gender, study period, geographic region, follow-up duration and nature of diabetes sample, were performed.

## Methods

### Search strategy and selection criteria

We systematically searched across the electronic databases Embase, MEDLINE, PsycInfo and Web of Science for articles published from inception to 20 December 2024. No restrictions were placed on language or geographic regions. The search strategy was developed by combining keywords, including depressive disorder, diabetes mellitus, mortality and complications. The full search strategy for each database is provided in Supplementary Table S2. The references of eligible articles were manually reviewed to identify additional relevant studies. Studies were included if they fulfilled the following criteria: (1) included patients diagnosed with diabetes mellitus, (2) reported on the risk of mortality or complications in patients with depression (defined as major depressive disorder ± dysthymia) and co-occurring diabetes compared to those with diabetes alone, and (3) were observational in design. Studies with ascertainment of mental disorders based on self-reported measures or unclear diagnostic criteria, which could not be mapped to DSM/ICD criteria, or classified based on symptoms but not disorders, were excluded. Publications addressing solely on specific population subgroups, or with a sample size of fewer than 50, were excluded. Conference abstracts, editorials, protocols and reviews that lack original data were also excluded. This meta-analysis was conducted and reported in accordance with the Preferred Reporting Items for Systematic Reviews and Meta-analyses (PRISMA) (Supplementary Table S1). The study protocol was registered prospectively with PROSPERO (CRD42024595145). Since this research was based on previously published literature, no ethical approval or patient consent was required.

### Study selection and data extraction

Duplicates among different databases were identified and removed, after which titles and abstracts were screened for relevant studies. Full texts of potentially eligible publications identified at screening were evaluated by two reviewers (M.T.H.H. and W.C.Y.C.) for inclusion. Disagreement was resolved through discussions or consultation with the supervisor (L.L.W.T.). Reasons for exclusion at this stage were documented. Data extraction included the following information: first author’s surname, year of publication, geographic region, study design, study period, types of diabetes, nature of diabetes sample (incident or prevalent), sample size of patients with depression and diabetes, sample size of patients with diabetes only, percentage of male in both groups, age at baseline with its standard deviation in both groups, outcome measure, cumulative incidence of outcome, risk estimates with 95% confidence intervals (CI), methods to ascertain depression, depression diagnostic system, inclusion of pre-existing depression, methods to ascertain diabetes and mortality/complication outcome, follow-up duration and matching and/or adjustment variables.

Risk estimates, when not reported numerically in the report, were extracted from the figures using WebPlotDigitizer (https://automeris.io/WebPlotDigitizer) (De Prisco *et al.*, 2024). For studies reporting outcome measures at multiple time points, the outcomes at the latest follow-up time point were selected. When several adjusted risk estimates were reported for the same outcome, the most comprehensively adjusted estimate was selected. Diabetes complications were categorized in broad terms as microvascular complications and macrovascular complications (Zheng *et al.*, 2018). To categorize specific types of diabetes complications, the Diabetes Complications Severity Index (DCSI) was adopted (Young *et al.*, 2008), which encompasses seven categories of complications, including cardiovascular, cerebrovascular, peripheral vascular, nephropathy, neuropathy, retinopathy and metabolic complications. Metabolic complications in DCSI encompass ketoacidosis, hyperosmolar state and other coma. Risk estimates were reported with 95% CI, with greater than one denoting a statistically significant increase in risk of mortality/complications in patients with depression. Risk-of-bias was independently assessed by two reviewers (M.T.H.H. and W.C.Y.C.) using the Newcastle-Ottawa Scale (NOS) (Wells *et al.*, 2009).

### Statistical analysis

Data synthesis was performed quantitatively. Odds ratio (OR), hazard ratio (HR) and incidence rate ratio (IRR) were treated as equivalent measures of risk to maximize data inclusion, in line with prior meta-analyses on mortality in mental disorders (Chen *et al.*, 2024; Ronaldson *et al.*, 2024). The term ‘relative risk’ (RR) is used thereafter. The analyses were also restricted to risk estimates reported by HR. The extracted risk estimates were log-transformed to achieve a normalized distribution, and their CIs were converted to standard errors (SE). The converted risk ratios and SE were then used to generate pooled estimates. As the included studies were conducted over different time periods and regions, substantial heterogeneity was anticipated across studies, and thus, random-effects meta-analysis was selected. Heterogeneity was evaluated using the *I*^2^ statistic (Higgins *et al.*, 2003), and Cochran’s *Q*-test was performed to assess the statistical significance of the heterogeneity across studies. Results were displayed graphically by Forest plot, presenting pooled estimates with 95% CIs and weight. When multiple risk estimates by subgroup were provided, the risk estimates were combined to derive a single estimate per study (with reference to van Dooren *et al.*, 2013), before they were pooled into a summary estimate across studies. Publication bias was examined visually through funnel plot and statistically by Egger’s regression asymmetry test, with *P* < 0.1 considered significant. Synthesized estimates subject to publication bias were further corrected for using Duval and Tweedie’s trim-and-fill procedure. Two sets of sensitivity analyses were conducted, including (1) restricting the analyses to studies classified as fair or good quality, and (2) leave-one-out analysis. Subgroup analyses were performed, stratified by pre-specified study-level characteristics, including the nature of the diabetes sample (incident or prevalent), the study period (categorized into ‘after year 2010’ or ‘before or in year 2010’ based on the middle year of the cohort data collection) and geographic regions. Univariate meta-regression was performed for age, gender (i.e., percentage male) and follow-up duration. Statistical analyses were performed in R (version 4.0.2) with *metafor* package. For all analyses, except Egger’s test, *P* < 0.05 was considered statistically significant.

## Results

### Study selection

**Figure 1.**
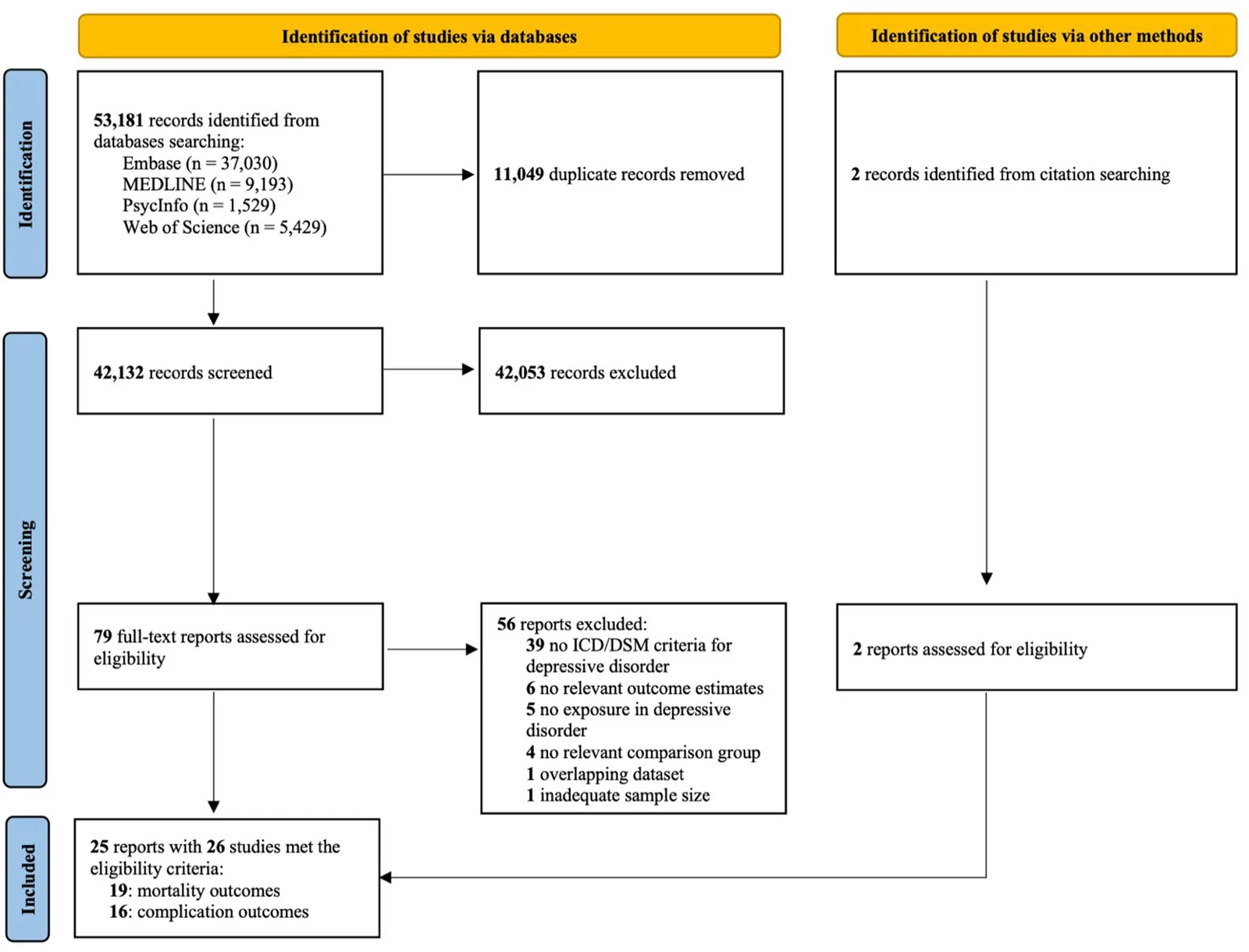
PRISMA flow diagram for study selection. Figure 1 long description.

The PRISMA flow diagram showing the screening and identification of studies is shown in Figure 1. A total of 53,181 records were identified in the literature search, and after removal of duplicates, 42,132 remained. After screening titles and abstracts, 79 full-text articles underwent full-text review. Of these, 56 were excluded due to: the exposure was not depressive disorder (*n* = 5), there were no DSM/ICD criteria for depressive disorder (*n* = 39), the comparison group was not patients with diabetes alone (*n* = 4); the study did not report relevant outcomes (*n* = 6), overlapping data (*n* = 1) and inadequate sample size (*n* = 1). An additional two records were identified from citation searching. Reasons for study exclusion are shown in Supplementary Table S3. Twenty-six studies met the inclusion criteria and were included in the meta-analysis.

### Study characteristics

Of these 26 studies, 19 studies were included in the meta-analysis for mortality outcomes and 16 studies for complication outcomes. As shown in Table 1, studies were conducted in the South Korea (*n* = 6), United States (*n* = 6), United Kingdom (*n* = 4), Taiwan (*n* = 3), Denmark (*n* = 2), Hong Kong (*n* = 2), Australia (*n* = 1), Canada (*n* = 1) and Spain (*n* = 1), which belonged to four continents including Asia (*n* = 11), Europe (*n* = 7), North America (*n* = 7) and Australia (*n* = 1).

**Table 1.** Characteristics of the included studies Table 1 long description.

							Mean age at baseline ± SD		Sample size			
Author ^a^(year)	Geographic location (Continent)	Study design	Study period	Type of diabetes	Incident/prevalent diabetes ^b^	Pre-existing DEP ^c^	DEP ^d^	Control ^d^	DEP ^d^(% male)	Control ^d^(% male)	Ethnicity	Outcome variables of interest
Ho *et al*., (2025a)	Hong Kong(Asia)	Cohort/longitudinal	2002−2021	Type 1 Type 2	Incident	Yes	61.8 ± 13.4	62.5 ± 13.7	12,175 (29.9%)	117,958 (31.0%)	NA	All-cause mortality, cardiovascular complications, cerebrovascular complications, peripheral vascular complications, nephropathy, neuropathy, retinopathy and metabolic complications
Li *et al*., (2025)	United Kingdom (Europe)	Cohort/longitudinal	2006−2022	Type 2	Prevalent	No	NA	NA	1,336 (49.4%)	12,370 (62.3%)	White (92.3%), others (7.1%)	All-cause mortality, CVD-specific mortality, cardiovascular complications, cerebrovascular complications, peripheral vascular complications, nephropathy, neuropathy and retinopathy
Kim *et al*., (2024)	South Korea (Asia)	Cohort/longitudinal	2009−2020	Type 1	Incident	Yes	NA	NA	2,030 (NA)	8,630 (NA)	NA	All-cause mortality, macrovascular complications
Scheuer *et al*., (2024)	Denmark (Europe)	Cohort/longitudinal	1996−2020	Type 1	Incident	No	NA	48.6 ± 18.7	1,186 (NA)	18,486 (60%)	NA	Metabolic complications
				Type 2			NA	63.0 ± 13.4	25,437 (NA)	378,480 (56.5%)	NA	
Yoo *et al*., (2023)	South Korea (Asia)	Cohort/longitudinal	2009−2018	Type 1	Prevalent	No	61.9 ± 11.0	56.2 ± 12.2	294,550 (41.5%)	2,152,836 (63.8%)	NA	Cardiovascular complications
				Type 2								
Fleetwood *et al*., (2023)	Scotland (Europe)	Cohort/longitudinal	2004−2019	Type 2	Incident	Yes	58.9 ± 12.7	60.8 ± 13.4	7,903 (40.3%)	248,140 (56.9%)	White (90.5%), ethnic minority (4.2%) and missing (5.3%)	All-cause mortality, macrovascular complications
Bae *et al*., (2022)	South Korea (Asia)	Cohort/longitudinal	2009−2018	Type 1 Type 2	Prevalent	Yes	62.4 ± 11.0	56.4 ± 12.3	313,773 (40.1%)	2,198,917 (63.0%)	NA	Cardiovascular complications
Kim *et al*., (2022)	South Korea (Asia)	Cohort/longitudinal	2009−2018	Type 2	Prevalent	No	60.3 ± 11.3	55.9 ± 12.2	88,903 (37.0%)	2,138,491 (62.2%)	NA	All-cause mortality, CVD-specific mortality, cardiovascular complications and cerebrovascular complications
Scheuer *et al*., (2022)	Denmark (Europe)	Cohort/longitudinal	1996−2018	Type 2	Prevalent	No	NA	NA	21,657 (NA)	341,523 (NA)	NA	Cardiovascular complications, peripheral vascular complications, nephropathy and retinopathy
Lunghi *et al*., (2021)	Canada (North America)	Nested case-control/longitudinal	2000−2008	Type 2	Incident	No	NA	NA	4,127 (NA)	144,842 (NA)	NA	All-cause mortality and CVD-specific mortality
Jung *et al*., (2021)	South Korea (Asia)	Cohort/longitudinal	2009−2017	Type 2	Prevalent	No	63.3 ± 10.6	56.5 ± 11.8	19,340 (37.4%)	2,166,979 (62.7%)	NA	All-cause mortality, cardiovascular complications and cerebrovascular complications
Wu *et al*., (2020)	Taiwan (Asia)	Cohort/longitudinal	2001−2014	Type 1 Type 2	Incident	Yes	52.6 ± 12.5	52.6 ± 12.5	38,537 (39.6%)	154,148 (39.6%)	NA	All-cause mortality, CVD-specific mortality, macrovascular complications and microvascular complications
Lee *et al*., (2020)	Taiwan (Asia)	Cohort/longitudinal	1999−2013	Type 1 Type 2	Incident	Yes	NA	NA	26,746 (42.5%)	106,853 (42.5%)	NA	Metabolic complications
Guerrero Fernández de Alba *et al*., (2020)	Spain (Europe)	Cohort/longitudinal	2012−2015	Type 2	Prevalent	No	NA	69.8 ± 12.2	8,628 (30.0%)	51,335 (57.9%)	NA	All-cause mortality
Chiang *et al*., (2020)	Taiwan (Asia)	Cohort/longitudinal	2001−2011	Type 2	Prevalent	No	NA	NA	628 (NA)	12,950 (NA)	NA	All-cause mortality
	United Kingdom (Europe)	Cohort/longitudinal	2006−2018	Type 2	Prevalent	No	NA	NA	1,643 (NA)	1,918 (NA)	NA	All-cause mortality
Jeong *et al*., (2017)	South Korea (Asia)	Cohort/longitudinal	2003−2013	Type 2	Prevalent	No	61.5 ± 10.8	59.1 ± 11.5	39,287 (39.2%)	1,003,802 (52.5%)	NA	All-cause mortality
Novak *et al*., (2016)	United States (North America)	Cohort/longitudinal	2004−2013	Type 2	Prevalent	No	61 ± 11	65 ± 11	340,806 (95%)	592,405 (97%)	White (78%), African American (17%), Hispanic (3%) and other race (2%)	Cardiovascular complications, cerebrovascular complications and nephropathy
Lynch *et al*., (2014)	United States (North America)	Cohort/longitudinal	2002−2006	Type 2	Prevalent	No	NA	NA	79,489 (NA)	546,414 (NA)	NA	All-cause mortality
Ting *et al*., (2013)	Hong Kong (Asia)	Cohort/longitudinal	1996−2009	Type 2	Prevalent	No	NA	NA	153 (21.6%)	7,682 (47.0%)	NA	All-cause mortality, macrovascular complications and cerebrovascular complications
Winkley *et al*., (2012)	United Kingdom (Europe)	Cohort/longitudinal	2001−2006	Type 1 Type 2	Prevalent	No	59.4 ± 14.6	63.3 ± 13.4	82 (59.8%)	171 (65.5%)	White (70.7%) and Black/Asian/other (29.3%)	All-cause mortality
Mai *et al*., (2011)	Western Australia (Australia)	Cohort/longitudinal	1990−2006	Type 1 Type 2	Prevalent	No	NA	NA	835 (NA)	26,626 (43.8%)	NA	All-cause mortality
Williams *et al*., (2011)	United States (North America)	Cohort/longitudinal	2000−2004	Type 1 Type 2	Prevalent	No	64.3 ± 11.7	67.2 ± 10.7	84,984 (95.9%)	446,989 (97.7%)	White (78.7%), Black (11.6%), Hispanic (5.0%), Asian/Pacific Islander (0.8%), Native American (1.7%), and unknown (2.3%)	Peripheral vascular complications
Katon *et al*., (2008)	United States (North America)	Cohort/longitudinal	2005−2007	Type1 Type2	Prevalent	Yes	71.0 ± 12.5	76.4 ± 8.2	1,657 (43.0%)	9,047 (58.7%)	White (90.4%), Black (5.5%), Hispanic (3.3%), and other (0.8%)	All-cause mortality
Richardson *et al*., (2008)	United States (North America)	Cohort/longitudinal	1996−2006	Type 2	Prevalent	No	56.0	61.9	806 (100%)	13,694 (100%)	Non-Hispanic white (62.5%), non-hispanic black (29.2%), and other (8.3%)	All-cause mortality
Clouse *et al*., (2003)	United States (North America)	Cohort/longitudinal	1982−1992	Type 1Type 2	Prevalent	No	47.0 ± 12.2	39.8 ± 16.3	16 (0%)	60 (0%)	NA	Cardiovascular complications

CVD = cardiovascular disease, MDD = major depressive disorder; NA = not available; N/A = not applicable; SD = standard deviation.

a The last name of the first author of the publication, in reverse chronological order of the date of publication.

b Refers to whether the study estimating the risk of mortality or complications based on an incident diabetes sample (i.e., patients with first diagnosed diabetes at the start of follow-up period) or a prevalent diabetes sample (i.e., patients with onset of diabetes mellitus prior to the start of follow-up period).

c Refers to whether the study examined the association by using pre-existing depressive disorder (i.e., depressive disorder that precedes diabetes) to delineate the temporal relationship of the association.

d DEP refers to people with depression and co-occurring diabetes mellitus (i.e., depression-diabetes group), and control refers to people with diabetes only (i.e., diabetes-only group).

Study periods spanned from 1982 to 2022 and were categorized into ‘after 2010’ (*n* = 10) or ‘before or in 2010’ (*n* = 16). Almost all studies used cohort design, with one adopting a nested case-control design. Seven studies focused on patients with incident diabetes, and 19 studies on patients with prevalent diabetes. Additional characteristics of individual studies are summarized in Supplementary Table S4. Twenty-four of the 26 studies ascertained the depression diagnosis by identifying diagnostic codes (ICD or DSM) in a health record database, while the remaining two studies used diagnostic interview (Clouse *et al.,* 2003; Winkley *et al.,* 2012) (Supplementary Table S4). The covariates of the individual studies are summarized in Supplementary Table S5, and the outcome estimates are summarized in Supplementary Tables S6 and S7. Of these 26 studies, the quality assessment showed that 25 were of fair to good quality, and 1 was of poor quality. It indicated that the majority of all studies were at low risk of bias. The corresponding NOS scores for each of the included studies on mortality and complication outcomes are presented in Supplementary Tables S8 and S9, respectively.

### Meta-analysis and stratified analyses of mortality outcomes

**Figure 2.**
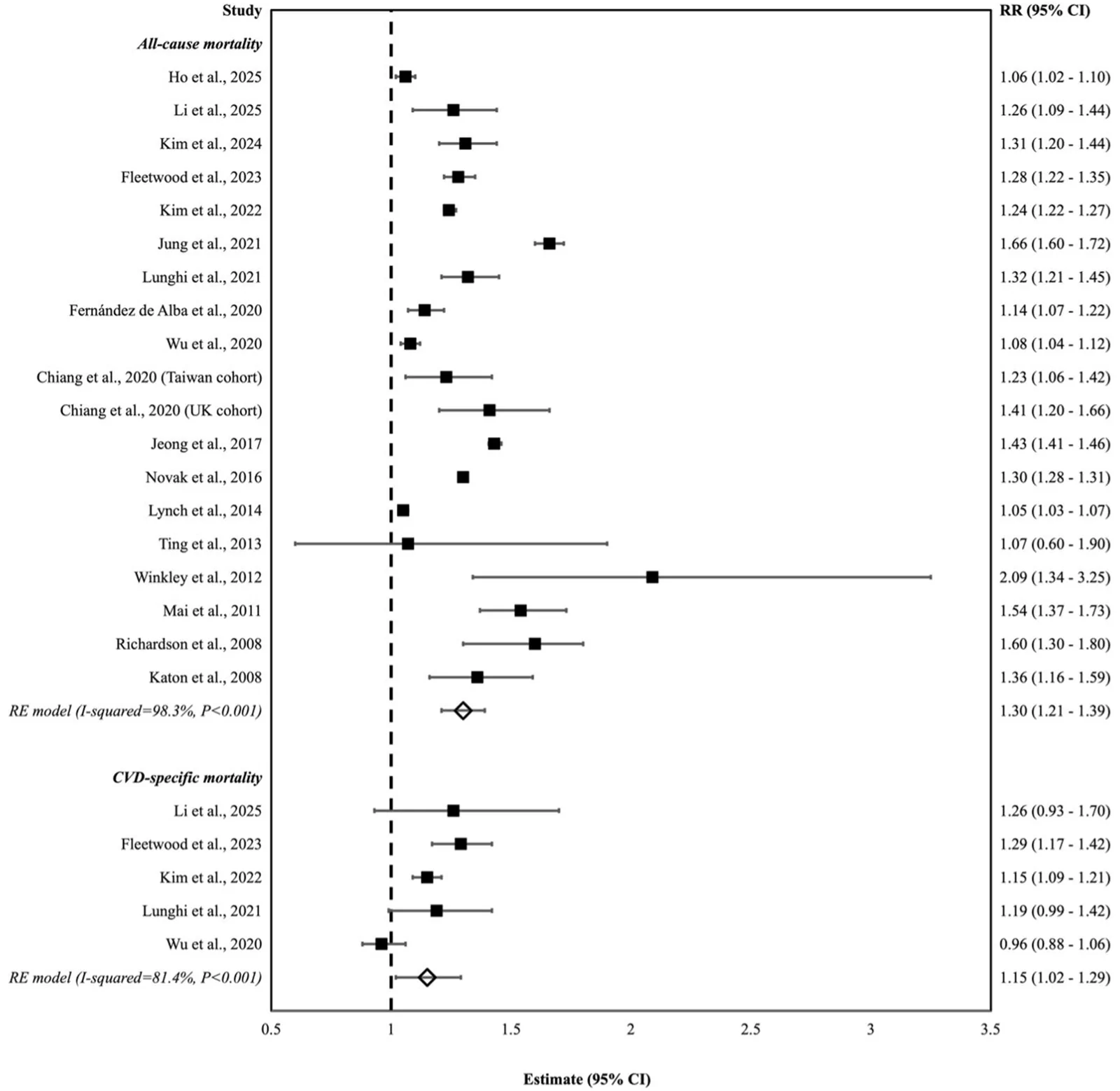
Forest plot for risk of all-cause mortality and CVD-specific mortality in diabetes patients with vs without depression. Studies were arranged in reverse chronological order (first author and their colleagues, year of publication). Figure 2 long description.

The risk estimates for all-cause and CVD-specific mortality are presented in Figure 2. The pooled estimate showed that the depression-diabetes group was associated with increased risk of all-cause mortality (RR 1.30 [95% CI: 1.21–1.39]), with significant heterogeneity (*Q*_18_ = 983.2, *P* < 0.001, *I*^2^ = 98.3%, *k* = 19). Visual inspection of the funnel plot (Supplementary Figure S1) and Egger’s test (*z* = 1.49, *P* = 0.137) suggested no publication bias. The synthesized estimate remained consistent when studies identified as of poor quality were excluded (1.29 [1.20–1.38]; *Q*_17_ = 769.0, *P* < 0.001, *I*^2^ = 98.0%, *k* = 18) and in leave-one-out analysis (1.27–1.32). Among the 19 studies assessing the all-cause mortality risk, 16 used HRs, 2 used ORs and 1 used IRRs. The synthesized estimate was (1.30 [1.20–1.40]); *Q*_15_ = 961.7, *P* < 0.001, *I*^2^ = 98.6%, *k* = 16) when the analysis was restricted to HR. Similarly, the pooled estimates showed that the depression-diabetes group was associated with increased risk of CVD-specific mortality (1.15 [1.02–1.29]; *Q*_4_ = 20.1, *P* < 0.001, *I*^2^ = 81.4%, *k* = 5). No evidence of publication bias was found in Egger’s test (*z* = 0.56, *P* = 0.578). Among the five studies assessing the CVD-specific mortality risk, four used HRs and one used ORs. The pooled estimate was (1.14 [0.99–1.31]); *Q*_3_ = 19.9, *P* < 0.001, *I*^2^ = 87.1%, *k* = 4) when the analysis was restricted to HR, and remained consistent in leave-one-out analysis (1.11–1.20).

Stratified analyses of all-cause mortality by geographic regions, study period and nature of diabetes are summarized in Table 2. Significant heterogeneity was observed in the risk of all-cause mortality and its stratified subgroups. In stratified analyses of all-cause mortality by geographic regions, the risk of all-cause mortality was higher in diabetes patients with depression than their non-depressed counterparts in Asia (1.27 [1.13–1.42]; *Q*_7_ = 513.5, *P* < 0.001, *I*^2^ = 98.7%, *k* = 8), in Europe (1.28 [1.16–1.41]; *Q*_4_ = 15.6, *P* = 0.004, *I*^2^ = 74.4%, *k* = 5), in North America (1.30 [1.14–1.48]; *Q*_4_ = 366.7, *P* < 0.001, *I*^2^ = 99.8%, *k* = 5) and in Australia (1.54 [1.37–1.73]) (Supplementary Figure S2). No significant group differences in mortality risk were detected for geographic regions (between-group *P* = 0.930). When stratified by study period, the risk in patients from studies conducted after 2010 (1.28 [1.16–1.41]; *Q*_7_ = 316.7, *P* < 0.001, *I*^2^ = 97.0%, *k* = 8) and from studies conducted before or in 2010 (1.32 [1.20–1.45]; *Q*_10_ = 666.0, *P* < 0.001, *I*^2^ = 98.7%, *k* = 11) were significantly higher in depression-diabetes group relative to the comparison group (Supplementary Figure S3). No significant group differences in mortality risk were detected for study period (between-group *P* = 0.298). When stratified by nature of diabetes, the risk of patients with prevalent diabetes (1.34 [1.24–1.46]; *Q*_13_ = 815.0, *P* < 0.001, *I*^2^ = 98.6%, *k =* 14) and those with incident diabetes (1.20 [1.09–1.32]; *Q*_4_ = 61.4, *P* < 0.001, *I*^2^ = 94.0%, *k* = 5) were significantly higher in the depression-diabetes group relative to the comparison group (Supplementary Figure S4). No significant group differences in mortality risk were detected for incident and prevalent diabetes status (between-group *P* = 0.116). Univariate meta-regression analyses showed a significant association between the percentage of male gender and the risk of depression-associated all-cause mortality in diabetes patients (β = 0.004 [0.003–0.008]; *Q_m_* = 4.44, *P* = 0.035), but not the study mean age at baseline of both groups (*Q_m_* = 0.19, *P* = 0.663) or the overall follow-up duration (*Q_m_* = 0.4, *P* = 0.517).

**Table 2.** Random-effects meta-analytic models showing mortality outcomes and stratified analyses Table 2 long description.

Mortality risks	No. of studies	RR (95% CI)	*I*^2^	*P* value ^a^	Between-group *P* value ^b^
All-cause mortality	19	1.30 (1.21−1.39)	98.3%	**<0.001**	
By geographic regions					0.930
Asia	8	1.27 (1.13−1.42)	98.7%	**<0.001**	
Europe	5	1.28 (1.16−1.41)	74.4%	**0.004**	
North America	5	1.30 (1.14−1.48)	98.8%	**<0.001**	
By study period					0.298
Before or in 2010	11	1.32 (1.20−1.45)	98.7%	**<0.001**	
After 2010	8	1.28 (1.16−1.41)	97.0%	**<0.001**	
By nature of diabetes					0.116
Incident	5	1.20 (1.09−1.32)	94.0%	**<0.001**	
Prevalent	14	1.34 (1.24−1.46)	98.6%	**<0.001**	
CVD-specific mortality	5	1.15 (1.02−1.29)	81.4%	**0.018**	

CI = confidence interval; CVD = cardiovascular disease; N/A = not applicable; RR = relative risk.

a *P* value for the comparison between depression-diabetes group versus diabetes-only group.

b *P* value for between-group comparison.

### Meta-analysis and stratified analyses of complication outcomes

**Figure 3.**
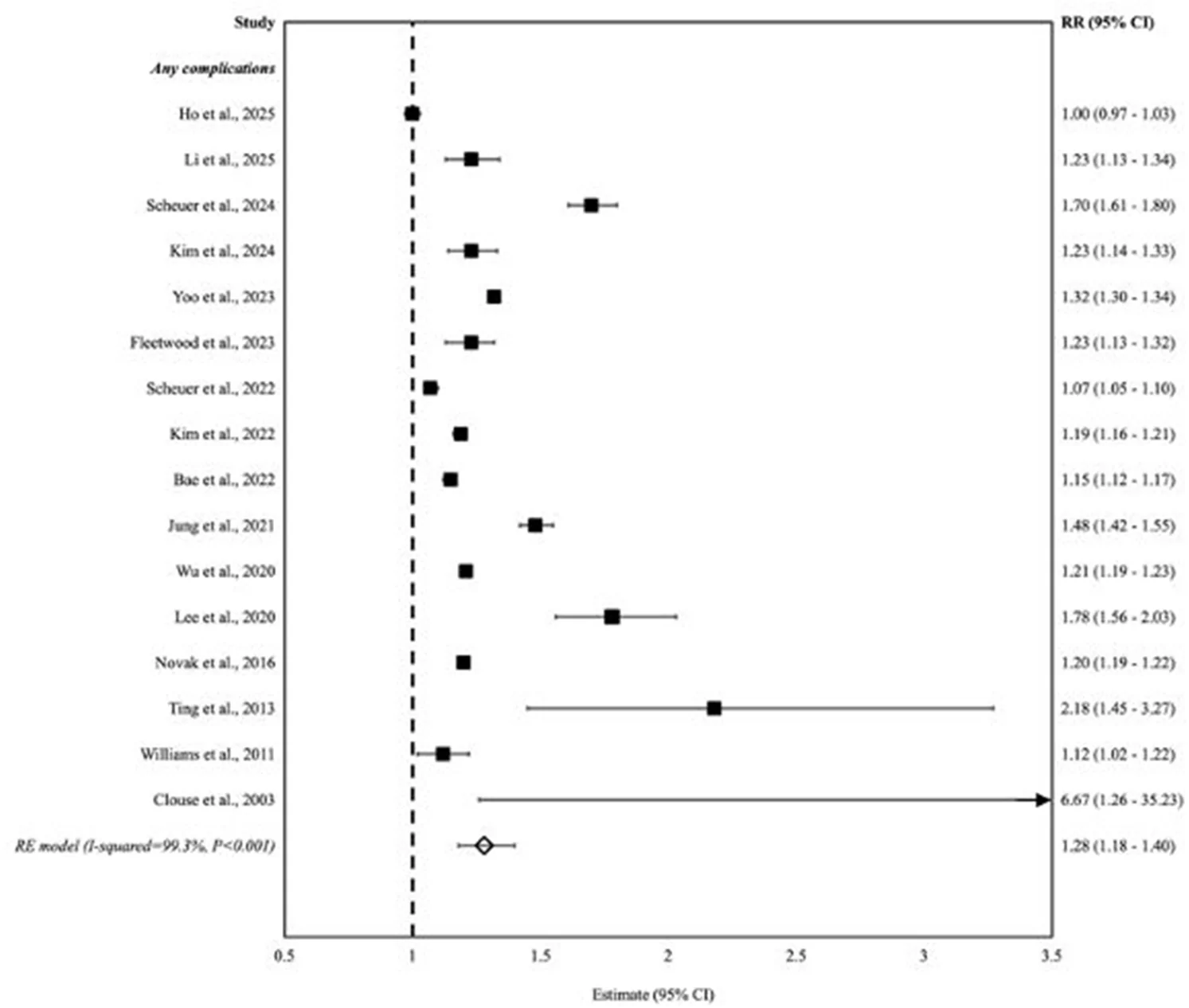
Forest plot for risk of any complications in diabetes patients with vs without depression. Studies were arranged in reverse chronological order (first author and their colleagues, year of publication). Figure 3 long description.

The synthesized estimate showed that the depression-diabetes group was associated with an increased risk of any complications (1.28 [1.18–1.40]) relative to the diabetes-only group, with significant heterogeneity (*Q*_15_ = 686.4, *P* < 0.001, *I*^2^ = 99.3%, *k* = 16) (Fig. 3). Publication bias was indicated by the asymmetrical funnel plot (Supplementary Figure S5) and a significant result of Egger’s test (*z* = 3.30, *P* = 0.001). The result remained robust after the trim-and-fill procedure was applied to correct for publication bias (1.28 [1.18–1.40]). Among the 16 studies assessing the complication risk, 14 used HRs and 2 used IRRs. The pooled estimate was (1.27 [1.17–1.38]; *Q*_13_ = 434.5, *P* < 0.001, *I*^2^ = 99.0%, *k* = 14) when the analysis was restricted to HR and remained consistent in leave-one-out analysis (1.25–1.31).

Stratified analyses of complication risk by geographic regions, study period, nature of diabetes and complication subtypes are summarized in Table 3. In stratified analyses by types of complications, the depression-diabetes group was associated with a higher rate of macrovascular complications (1.23 [1.15–1.31]; *Q*_13_ = 469.6, *P* < 0.001, *I*^2^ = 98.2%, *k* = 14) than the diabetes-only group, but not microvascular complications (1.08 [0.97–1.19]; *Q*_4_ = 222.1, *P* < 0.001, *I*^2^ = 98.3%, *k* = 5) (Supplementary Figure S6). Relative to diabetes-only group, depression-diabetes group was associated with a higher rate of metabolic complications (1.63 [1.33–1.99]; *Q*_2_ = 10.1, *P* = 0.006, *I^2^* = 83.7%, *k* = 3) and cardiovascular complications (1.20 [1.11–1.29]; *Q*_8_ = 255.5, *P* < 0.001, *I*^2^ = 97.7%, *k* = 9), and lower likelihood of retinopathy (0.84 [0.76–0.94]; *Q*_2_ = 4.3, *P* < 0.001, *I*^2^ = 54.7%, *k* = 3), although cerebrovascular complications (1.36 [0.99–1.87]; *Q*_5_ = 141.5, *P* < 0.001, *I*^2^ = 99.6%, *k* = 6), nephropathy (1.09 [0.93–1.27]; *Q*_3_ = 94.4, *P* < 0.001, *I*^2^ = 98.4%, *k* = 4) and peripheral vascular complications (0.97 [0.79–1.18]; *Q*_3_ = 19.7, *P* < 0.001, *I*^2^ = 86.3%, *k* = 4) were not associated with significant rate differences (Supplementary Figure S7). In stratified analyses by nature of diabetes, an increased risk of complications was observed in depression patients with incident diabetes (1.32 [1.11–1.58]; *Q*_5_ = 327.2, *P* < 0.001, *I*^2^ = 99.0%, *k* = 6) and prevalent diabetes (1.24 [1.15–1.34]; *Q*_9_ = 354.6, *P* < 0.001, *I*^2^ = 98.7%, *k =* 10) (Supplementary Figure S8). No significant group differences in complication risk were detected for incident and prevalent diabetes status (between-group *P* = 0.604). In stratified analyses by geographic regions, an increased risk of complications was observed in depression patients with diabetes in Asia (1.31 [1.15–1.48]; *Q*_8_ = 420.8, *P* < 0.001, *I*^2^ = 99.5%, *k* = 9), in Europe (1.29 [1.06–1.56]; *Q*_3_ = 230.6, *P* < 0.001, *I*^2^ = 98.1%, *k =* 4) and in North America (1.18 [1.11–1.25]; *Q*_2_ = 6.3, *P* < 0.001, *I*^2^ = 35.8%, *k* = 3) (Supplementary Figure S9). No significant group differences in complication risk were detected for geographic regions (between-group *P* = 0.850). In stratified analyses by study period, a higher risk of complications was observed in studies with study period after 2010 (1.22 [1.13–1.32]; *Q*_7_ = 380.0, *P* < 0.001, *I*^2^ = 98.2%, *k* = 8) and before or in 2010 (1.40 [1.16–1.69]; *Q*_7_ = 292.4, *P* < 0.001, *I*^2^ = 99.6%, *k =* 8) (Supplementary Figure S10). No significant group differences in complication risk were detected after 2010 and before or in 2010 (between-group *P* = 0.214). Univariate meta-regression analyses found no significant associations between risk of any complications and study mean age at baseline of both groups (*Q_m_* = 2.90, *P* = 0.09), percentage of male gender of both groups (*Q_m_* = 0.79, *P* = 0.375) and overall follow-up duration (*Q_m_* = 0.1, *P* = 0.722).

**Table 3.** Random-effects meta-analytic models showing complication outcomes and stratified analyses Table 3 long description.

Complication outcomes	No. of studies	RR (95% CI)	*I*^2^	*P* value ^a^	Between-group *P* value ^b^
Any complications	16	1.28 (1.18−1.40)	99.3%	**<0.001**	
By geographic regions					0.850
Asia	9	1.31 (1.15−1.48)	99.5%	**<0.001**	
Europe	4	1.29 (1.06−1.56)	98.1%	**0.011**	
North America	3	1.18 (1.11−1.25)	35.8%	**<0.001**	
By study period					0.214
Before or in 2010	8	1.40 (1.16−1.69)	99.6%	**<0.001**	
After 2010	8	1.22 (1.13−1.32)	98.2%	**<0.001**	
By nature of diabetes					0.604
Incident	6	1.32 (1.11−1.58)	99.0%	**0.001**	
Prevalent	10	1.24 (1.15−1.34)	98.7%	**<0.001**	
Complication subtypes					
Macrovascular complications	14	1.23 (1.15−1.31)	98.2%	**<0.001**	
Microvascular complications	5	1.08 (0.97−1.19)	98.3%	0.164	
Metabolic complications	3	1.63 (1.33−1.99)	83.7%	**<0.001**	
Cardiovascular complications	9	1.20 (1.11−1.29)	97.7%	**<0.001**	
Cerebrovascular complications	6	1.36 (0.99−1.87)	99.6%	0.060	
Peripheral vascular complications	4	0.97 (0.79−1.18)	86.3%	0.747	
Nephropathy	4	1.09 (0.93−1.27)	98.4%	0.303	
Retinopathy	3	0.84 (0.76−0.94)	54.7%	**0.002**	

*Note:* CI: confidence interval; RR: relative risk.

a *P* value for the comparison between depression-diabetes group versus diabetes-only group.

b *P* value for between-group comparison.

## Discussion

To our knowledge, this is the largest systematic review and meta-analysis to summarize the risk of mortality and complications in patients with depressive disorder and co-occurring diabetes relative to diabetes-only patients, to investigate the associations between depression and a broad range of diabetes-related outcomes and to evaluate the impact of potential moderators on the differential mortality and complication risk using subgroup analyses and meta-regression on an array of study-level characteristics.

The main analyses of mortality outcome indicated that patients with depression and comorbid diabetes exhibited 30% increased risk of all-cause mortality relative to non-depressed counterparts. Our result was consistent with that of Hofmann *et al.*, (2013), which reported a 49% increased mortality risk in their meta-analysis. Albeit more modest, the all-cause mortality risks across studies were consistently elevated, with robust pooled estimates confirmed by the sensitivity analyses. Our findings also suggested that a greater percentage of males may increase the depression-associated all-cause mortality risk in patients with diabetes, suggesting that male patients could be at greater risk. Notably, such increased mortality risk was consistent across Asia, Europe and North America (i.e., 27%, 28% and 30% increased risk, respectively), in studies conducted before and after 2010 (i.e., 32% and 28% increased risk, respectively), and regardless of incident or prevalent diabetes status, suggesting that the mortality gap was present over distinct regions and have persisted over time. A meta-analysis focusing on schizophrenia-spectrum disorder (SSD) and bipolar disorder (BD) had demonstrated a 77% higher all-cause mortality risk (Ho *et al.*, 2025b) in this respect. As appeared across the diagnoses of depression, SSD and BD, the influence of mental disorders on the clinical outcomes of diabetes might be relatively transdiagnostic, and the differential mortality risk points to the importance of investigating the attenuating or aggravating factors related to diabetes outcomes in depression.

The analyses of complication outcomes indicated that patients with depression and comorbid diabetes exhibited significantly higher risk of any complications relative to non-depressed counterparts, regardless of incident or prevalent diabetes status. Importantly, such increased complication risk was demonstrated in various geographic regions and has persisted over time, suggesting the need for interventions to reduce the health disparity in depression. Analyses of the major specific complications observed higher rates of metabolic and cardiovascular complications, and lower rates of retinopathy in the depression-diabetes group than in the diabetes-only group, albeit comparable rates of nephropathy, peripheral vascular complications and cerebrovascular complications. The markedly elevated risk of metabolic complications in the depression-diabetes group likely reflects acute glycemic fluctuations as characterized by events such as diabetic ketoacidosis and hyperosmolar hyperglycemic state. Although the available data did not allow us to isolate the contribution of each specific event type, it highlights the need for closer monitoring of glycemic instability, early detection of treatment non-adherence and difficulties in diabetes self-management that might lead to rapid metabolic decompensation in patients with depression and diabetes. Conversely, a lower or similar rate of vascular complications could be due to a competing risk model from premature death that precluded the emergence of advanced vascular complications. Alternatively, it might be due to under-recognition of diabetes complications, driven by inadequate understanding at the patient level and by disparities in diabetes treatment and monitoring at the clinician and system levels (Wagner *et al.*, 2026). While patients might present with acute-onset and distressing symptoms (e.g., coma and myocardial infarction) warranting clinical attention, suboptimal glycemic control could silently drive any hidden chronic complications (e.g., progressive visual blurring and kidney failure), and ultimately the mortality gap in the long term.

It is acknowledged that the association between depression and excess mortality among patients with co-occurring diabetes is complex (Hackett and Steptoe, 2017). Depressive symptoms, such as reduced motivation and negative cognitions, may affect patients’ self-management of diabetes, leading to delayed help-seeking, treatment non-adherence and unfavourable lifestyle behaviours (Berk *et al.*, 2023), thereby resulting in poor glycemic control and worse physical outcomes (Lustman *et al.*, 2000; Gonzalez *et al.*, 2008). Patients’ diabetes outcomes might also be adversely affected by healthcare disparities, with lower receipt of equitable diabetes care and suboptimal treatment (Wagner *et al.*, 2026). Accumulating evidence has also indicated shared pathophysiological mechanisms underlying both conditions, including hypothalamic-pituitary-adrenal axis hyperactivity, immune-inflammatory dysregulation and overlapping genetic and epigenetic factors (Vreeburg *et al.*, 2009; Stapelberg *et al.*, 2011). In addition, biological and behavioural factors that are connected with both depression and diabetes, such as obesity and sedentary lifestyles (Tabák *et al.*, 2014), could be implicated in the poorer clinical outcomes. Notably, evidence indicated that successful treatment for depression is able to enhance glycemic control (Croatto *et al.*, 2023), therefore highlighting that treating mental disorders through effective management can reduce their adverse effects on physical health outcomes (Dragioti *et al.*, 2023). Optimization of antidepressants in patients with comorbid depression and diabetes may additionally contribute to the reduction of all-cause mortality (Ho *et al.*, 2026). However, not all existing treatment strategies for patients with comorbid depression and diabetes are considered effective for glycemic control (van der Feltz-cornelis *et al.*, 2021). Identifying effective interventions is important, including integrated and targeted approaches (Petrak *et al.*, 2015), such as improving diabetes screening and management in psychiatric settings. Improving the provision of lifestyle advice and community support might mitigate the shared risk factors across depression and diabetes that drive the risk of adverse clinical outcomes. Precision psychiatry that aims to provide personalized diabetes care might identify patients with depression who are at risk of developing diabetes, and thus, pre-emptive interventions can be delivered to prevent the onset of diabetes in this at-risk population (Perry *et al.*, 2026).

Several limitations warrant consideration in interpreting the study results. First, the overall risk estimates should be interpreted with caution. To maximize data inclusion, we pooled ORs, HRs and IRRs into a single relative risk estimate, but these effect measures are not strictly equivalent, and ORs may overestimate relative risk when the outcomes are common. In the current analysis, the pooled risk estimates were dominated by HR, as extracted from the primary studies. The sensitivity analyses restricted to HRs are consistent with the pooled risk estimates and could minimize this concern. Second, significant heterogeneity was found across most meta-analytic estimates, and this heterogeneity could not be entirely accounted for by the stratified analyses based on study-level characteristics. The substantial heterogeneity likely reflects clinical and methodological diversity among the included studies. The presence of high heterogeneity as such could be attributed to different sociocultural contexts and health systems in distinct geographic regions with variable degrees of health literacy, health-seeking behaviour and medication prescribing practices in the diabetes population. Across studies, methodological variations with inconsistencies in the ascertainment of depression (e.g., differences in diagnostic thresholds in different health systems), different degrees of accuracy and representativeness of healthcare databases, differences in confounder adjustment (e.g., varying confounding variables including lifestyle factors, socioeconomic status and physical comorbidities), inconsistencies in the coding of diabetes or diabetes complications and the variations in depression and diabetes management over the study periods covering several decades might also account for the heterogeneity. At these high levels of heterogeneity, the pooled risks should be interpreted cautiously, noting that it represents a descriptive estimate of central tendency within a highly variable distribution of effects, rather than a precise population-level effect size. This indicates the need for future studies to standardize diagnostic and outcome ascertainment. Third, the vast majority of the included studies did not employ formal competing-risk regression methods to account for the competing risk of death when analysing outcomes of diabetes complications. This represents a systematic methodological limitation of the complication analyses, as Cox proportional hazards models used in the primary studies may overestimate complication incidence in the depression-diabetes group by failing to properly handle mortality as a competing event. The observed lower rates of retinopathy and comparable rates of nephropathy and peripheral vascular complications may therefore partly reflect this analytical limitation rather than true clinical differences. Future research should incorporate competing-risk methods to provide more robust estimates of complication risks in this population.

Fourth, there is a scarcity of data for subgroup analyses and meta-regression, and the results should be re-evaluated when more studies are conducted. Fifth, to minimize the confounding effects of other covariates on the associations between depression and diabetes outcomes, the most adjusted estimates were selected. However, such estimates might be overadjusted and no longer represent the real impact of depression on diabetes outcomes. Alternatively, although the effect of depression was isolated by selecting the fully-adjusted estimate, it might still be confounded by unmeasured or residual variables. Sixth, the included studies were geographically concentrated in high-income countries, predominantly the United States, the United Kingdom and South Korea, with the absence of evidence from low- and middle-income countries (LMICs) that bear a significant disease burden of depression and diabetes. Our findings may reflect health systems with relatively advanced infrastructure and greater capacity of diagnostic ascertainment, enabling the identification of depression and diabetes complications. Caution is warranted in generalizing the results to LMIC settings with variable patterns of healthcare services and quality of diabetes care. Future studies should incorporate findings from different geographic regions, particularly LMICs, to determine the extent to which these associations hold across regions. Seventh, the findings of several specific complications, including retinopathy, nephropathy, peripheral vascular complications and metabolic complications, were based only on a few studies and should be interpreted with caution. The limited number of contributing studies precludes robust conclusions about these specific complications and requires replications in larger pools of primary studies. Eighth, most studies focused on type 2 diabetes, with only two studies examining type 1 diabetes. Many studies included both type 1 and type 2 diabetes and did not delineate their analyses by diabetes subtypes, but their sample is likely to be predominantly type 2 diabetes. Due to the limited data on type 1 diabetes, we could not further evaluate the differential effect of depression on the physical health outcomes in relation to diabetes subtypes. Future studies should separately investigate type 1 diabetes, given its distinct pathophysiological mechanisms. Ninth, similar to many meta-analyses, publication bias was found, indicating the presence of unpublished literature. Notwithstanding, this meta-analysis confirms the adverse influence of depression on mortality and complications in patients with co-existing diabetes and provides preliminary targets to improve the clinical outcome in patients with depression in the context of diabetes.

In conclusion, our study demonstrated that patients with depression and co-occurring diabetes were associated with an elevated overall mortality risk compared to their non-depressed counterparts. Such an increased all-cause mortality risk was present across geographical regions and has persisted over time. Depression patients with comorbid diabetes were also associated with a higher risk of metabolic and cardiovascular complications, implying an overall poorer glycemic control that might eventually drive their earlier death. This study provided a comprehensive evidence synthesis that indicates an unmet treatment need for patients with depression to rectify their poorer diabetes-related outcomes. A multilevel interventional framework, including potential development of depression-specific risk prediction models for major diabetes complications, is needed for individualized risk estimation of diabetes-related outcomes, with consequent early interventions to minimize the avoidable physical morbidity and premature mortality in this vulnerable population.

## Supporting information

10.1017/S2045796026100882.sm001 Ho et al. supplementary material Ho et al. supplementary material

## Data Availability

The data that support the findings of this study are available from the corresponding author upon reasonable request.

## Supplementary material

The supplementary material for this article can be found at https://doi.org/10.1017/S2045796026100882.

## Figure 1 Long description

The flowchart illustrates the PRISMA process for study selection. It begins with the identification of studies via databases, listing 53,181 records identified from sources like Embase, MEDLINE, PsycINFO and Web of Science. After removing 11,049 duplicates, 42,132 records remain. Additionally, 2 records are identified from citation searching. During screening, 42,053 records are excluded, leaving 79 full-text reports assessed for eligibility. Of these, 56 reports are excluded for reasons such as not meeting DSM/ICD criteria for depressive disorder, irrelevant outcome estimates, incorrect comparison group, overlapping data and inadequate sample size. Two reports are assessed for eligibility from other methods. Finally, 25 reports with 26 studies meet the eligibility criteria, including 10 mortality outcomes and 16 complication outcomes.

Navigate back to Figure 1.

## Figure 2 Long description

Two stacked forest plots labeled “All-cause mortality” and “CVD-specific mortality,” each listing studies in a left column and showing square markers with horizontal confidence-interval lines aligned to a central vertical reference line at 1. All-cause mortality section: The right column header reads “RR (95 percent CI)”. The x-axis label reads “Estimate (95 percent CI)”, with tick labels 0.5, 1, 1.5, 2, 2.5, 3 and 3.5. The study rows and values shown are: Ho et al., 2025: 1.06 (1.02 - 1.10). Li et al., 2025: 1.26 (1.09 - 1.44). Kim et al., 2024: 1.31 (1.20 - 1.44). Fleetwood et al., 2023: 1.28 (1.22 - 1.35). Kim et al., 2022: 1.24 (1.22 - 1.27). Ung et al., 2021: 1.66 (1.60 - 1.72). Lughati et al., 2021: 1.32 (1.21 - 1.45). Fernandes de Abreu et al., 2020: 1.14 (1.07 - 1.22). Wu et al., 2020: 1.08 (1.04 - 1.12). Chiang et al., 2020 (Taiwan cohort): 1.23 (1.06 - 1.42). Chiang et al., 2020 (UK cohort): 1.41 (1.20 - 1.66). Jeong et al., 2017: 1.43 (1.41 - 1.46). Novak et al., 2016: 1.30 (1.28 - 1.31). Lynch et al., 2014: 1.05 (1.03 - 1.07). Ting et al., 2013: 1.07 (0.60 - 1.90). Winkley et al., 2012: 2.09 (1.34 - 3.25). Mai et al., 2011: 1.54 (1.37 - 1.73). Rikhardson et al., 2008: 1.60 (1.30 - 1.80). Katon et al., 2008: 1.36 (1.16 - 1.59). A pooled-effect row shows a diamond with: 1.30 (1.21 - 1.39). CVD-specific mortality section: The same right column header “RR (95 percent CI)” appears. The x-axis label remains “Estimate (95 percent CI)” with the same tick labels. The study rows and values shown are: Li et al., 2025: 1.26 (0.93 - 1.70). Fleetwood et al., 2023: 1.29 (1.17 - 1.42). Kim et al., 2022: 1.15 (1.09 - 1.21). Lughati et al., 2021: 1.19 (0.99 - 1.42). Wu et al., 2020: 0.96 (0.88 - 1.06). A pooled-effect row shows a diamond with: 1.15 (1.02 - 1.29).

Navigate back to Figure 2.

## Figure 3 Long description

Study A forest plot with a left column listing study names and years, a central plot area with square markers and horizontal confidence-interval lines and a right column labeled “RR (95 percent CI)”. A vertical dashed reference line is drawn at 1. The x-axis is labeled “Estimate (95 percent CI)”, ranging from 0.5 to 3.5 with tick marks at 0.5, 1, 1.5, 2, 2.5, 3 and 3.5. The plotted study estimates are shown as squares, each with a horizontal line for the confidence interval. Most squares are positioned to the right of the reference line at 1. The right column lists the following values: 1.30 (0.97 to 1.74) 1.23 (0.91 to 1.66) 1.23 (0.91 to 1.66) 1.23 (0.91 to 1.66) 1.32 (1.03 to 1.68) 1.23 (0.91 to 1.66) 1.32 (1.03 to 1.68) 1.23 (0.91 to 1.66) 1.39 (1.16 to 1.67) 1.35 (1.16 to 1.57) 1.35 (1.16 to 1.57) 1.35 (1.16 to 1.57) 1.21 (0.91 to 1.59) 1.80 (0.96 to 3.38) 1.20 (0.91 to 1.59) 2.10 (1.49 to 2.97) 1.28 (1.18 to 1.40) A diamond marker near the bottom represents the pooled estimate, aligned with 1.28 (1.18 to 1.40).

Navigate back to Figure 3.

## Table 1 Long description

Study characteristics for 23 longitudinal studies compare people with depression plus diabetes versus diabetes only, reporting location, design, years, diabetes type, incident or prevalent sampling, baseline age, sample sizes, sex distribution, ethnicity, and outcomes. Most studies are cohort designs spanning the 1990s through 2025, with many conducted in Asia and Europe and several in North America. Type 2 diabetes predominates, though multiple studies include both type 1 and type 2, and a few focus on type 1 only. Many samples are very large, including national cohorts with hundreds of thousands to millions of participants, while a few early studies are small. Baseline ages are typically in the late fifties to early seventies when reported, but age and sex data are frequently missing for some studies. Outcomes most often include all-cause mortality and cardiovascular outcomes, with additional reporting of cerebrovascular, peripheral vascular, nephropathy, neuropathy, retinopathy, and metabolic complications. Several studies explicitly assess whether depression predates diabetes, and both incident and prevalent diabetes cohorts are represented, which affects how temporality and risk estimates can be interpreted. Ethnicity is reported in only a subset of studies, commonly noting predominantly White samples in United Kingdom and United States cohorts, limiting cross-study comparability on demographic composition.

Navigate back to Table 1.

## Table 2 Long description

Random-effects meta-analysis estimates relative risk of mortality for people with both depression and diabetes compared with diabetes only, reporting study counts, confidence intervals, heterogeneity, and p values. All-cause mortality across 19 studies shows a relative risk of 1.30 with a confidence interval from 1.21 to 1.39 and very high heterogeneity at 98.3 percent, with a p value below 0.001. By region, relative risks are similar: Asia 1.27, Europe 1.28, and North America 1.30; the between-region comparison p value is 0.930, suggesting no meaningful difference by region. By study period, results are 1.32 for studies before or in 2010 and 1.28 for studies after 2010; the between-period p value is 0.298. By diabetes nature, incident diabetes shows 1.20 and prevalent diabetes shows 1.34; the between-group p value is 0.116. Cardiovascular disease-specific mortality across 5 studies shows a relative risk of 1.15 with a confidence interval from 1.02 to 1.29 and heterogeneity of 81.4 percent, with a p value of 0.018. Interpretation should note the consistently high heterogeneity, indicating substantial variation across studies even when pooled effects are statistically significant.

Navigate back to Table 2.

## Table 3 Long description

Random-effects meta-analysis results compare complication risks in people with both depression and diabetes versus diabetes alone, reporting relative risk, confidence intervals, heterogeneity, and p values. Overall, any complication is higher across 16 studies, with a relative risk of 1.28 and very high heterogeneity. By region, risks are similar in Asia, Europe, and North America, and the between-region difference is not statistically significant. By study period, risk is higher in studies before or in 2010 than after 2010, but the between-period difference is not statistically significant. By diabetes type, incident and prevalent diabetes show similar elevated risks, with no significant between-group difference. By subtype, macrovascular, metabolic, and cardiovascular complications show statistically significant increases, while microvascular, cerebrovascular, peripheral vascular complications, and nephropathy are not statistically significant. Retinopathy shows a statistically significant lower risk, and several subtype estimates have high heterogeneity, so results may vary notably across studies.

Navigate back to Table 3.
