# Supplementary material for: Risk of mortality and complications in people with depressive disorder and co-occurring diabetes mellitus: a systematic review and meta-analysis
Source: Epidemiol Psychiatr Sci. 2026 Sep 4;35:e49. doi: 10.1017/S2045796026100882 (PMC13735844; doi:10.1017/S2045796026100882)
Supplement: Supplementary file 1 [file S2045796026100882sup001.docx]

**Supplementary Materials**

**Table S1.** PRISMA checklist.

**Table S2.** Full search strategy in electronic databases.

**Table S3.** List of excluded studies and the reasons for exclusions.

**Table S4.** Additional characteristics of included studies.

**Table S5.** Matching variables and adjustment variables for the risk estimates in the included studies.

**Table S6.** Mortality outcome of included studies.

**Table S7.** Complication outcome of included studies.

**Table S8.** Newcastle-Ottawa Scale for assessing the quality of included studies for mortality outcomes in cohort studies and case-control studies.

**Table S9.** Newcastle-Ottawa Scale for assessing the quality of included studies for complication outcomes in cohort studies and case-control studies.

**Figure S1.** Funnel plot for publication bias in meta-analysis of risk of all-cause mortality in diabetes patients with versus without depression.

**Figure S2.** Forest plot for risk of all-cause mortality in diabetes patients with vs without depression stratified by geographic regions.

**Figure S3.** Forest plot for risk of all-cause mortality in diabetes patients with vs without depression stratified by study period.

**Figure S4.** Forest plot for risk of all-cause mortality in diabetes patients with vs without depression stratified by nature of diabetes sample.

**Figure S5.** Funnel plot for publication bias in meta-analysis of risk of any complications in diabetes patients with versus without depression.

**Figure S6.** Forest plot for risk of macrovascular and microvascular complications in diabetes patients with vs without depression.

**Figure S7.** Forest plot for risk of specific diabetes complications in diabetes patients with vs without depression.

**Figure S8.** Forest plot for risk of any complications in diabetes patients with vs without depression stratified by nature of diabetes.

**Figure S9.** Forest plot for risk of any complications in diabetes patients with vs without depression stratified by geographic regions.

**Figure S10.** Forest plot for risk of any complications in diabetes patients with vs without depression stratified by study period.

**Table S1.**

PRISMA checklist.

| Section and Topic | Item # | Checklist item | Location where item is reported |
| --- | --- | --- | --- |
| **TITLE** | | |  |
| Title | 1 | Identify the report as a systematic review. | Title page |
| **ABSTRACT** | | |  |
| Abstract | 2 | See the PRISMA 2020 for Abstracts checklist. | Abstract |
| **INTRODUCTION** | | |  |
| Rationale | 3 | Describe the rationale for the review in the context of existing knowledge. | Introduction |
| Objectives | 4 | Provide an explicit statement of the objective(s) or question(s) the review addresses. | Introduction |
| **METHODS** | | |  |
| Eligibility criteria | 5 | Specify the inclusion and exclusion criteria for the review and how studies were grouped for the syntheses. | Selection criteria |
| Information sources | 6 | Specify all databases, registers, websites, organisations, reference lists and other sources searched or consulted to identify studies. Specify the date when each source was last searched or consulted. | Search strategy |
| Search strategy | 7 | Present the full search strategies for all databases, registers and websites, including any filters and limits used. | Table S2 |
| Selection process | 8 | Specify the methods used to decide whether a study met the inclusion criteria of the review, including how many reviewers screened each record and each report retrieved, whether they worked independently, and if applicable, details of automation tools used in the process. | Study selection |
| Data collection process | 9 | Specify the methods used to collect data from reports, including how many reviewers collected data from each report, whether they worked independently, any processes for obtaining or confirming data from study investigators, and if applicable, details of automation tools used in the process. | Data extraction |
| Data items | 10a | List and define all outcomes for which data were sought. Specify whether all results that were compatible with each outcome domain in each study were sought (e.g. for all measures, time points, analyses), and if not, the methods used to decide which results to collect. | Data extraction |
|  | 10b | List and define all other variables for which data were sought (e.g. participant and intervention characteristics, funding sources). Describe any assumptions made about any missing or unclear information. | Data extraction |
| Study risk of bias assessment | 11 | Specify the methods used to assess risk of bias in the included studies, including details of the tool(s) used, how many reviewers assessed each study and whether they worked independently, and if applicable, details of automation tools used in the process. | Data extraction |
| Effect measures | 12 | Specify for each outcome the effect measure(s) (e.g. risk ratio, mean difference) used in the synthesis or presentation of results. | Statistical analysis |
| Synthesis methods | 13a | Describe the processes used to decide which studies were eligible for each synthesis (e.g. tabulating the study intervention characteristics and comparing against the planned groups for each synthesis (item #5)). | Statistical analysis |
|  | 13b | Describe any methods required to prepare the data for presentation or synthesis, such as handling of missing summary statistics, or data conversions. | Statistical analysis |
|  | 13c | Describe any methods used to tabulate or visually display results of individual studies and syntheses. | Statistical analysis |
|  | 13d | Describe any methods used to synthesize results and provide a rationale for the choice(s). If meta-analysis was performed, describe the model(s), method(s) to identify the presence and extent of statistical heterogeneity, and software package(s) used. | Statistical analysis |
|  | 13e | Describe any methods used to explore possible causes of heterogeneity among study results (e.g. subgroup analysis, meta-regression). | Statistical analysis |
|  | 13f | Describe any sensitivity analyses conducted to assess robustness of the synthesized results. | Statistical analysis |
| Reporting bias assessment | 14 | Describe any methods used to assess risk of bias due to missing results in a synthesis (arising from reporting biases). | Data extraction |
| Certainty assessment | 15 | Describe any methods used to assess certainty (or confidence) in the body of evidence for an outcome. | Statistical analysis |
| **RESULTS** | | |  |
| Study selection | 16a | Describe the results of the search and selection process, from the number of records identified in the search to the number of studies included in the review, ideally using a flow diagram. | Results |
|  | 16b | Cite studies that might appear to meet the inclusion criteria, but which were excluded, and explain why they were excluded. | Table S3 |
| Study characteristics | 17 | Cite each included study and present its characteristics. | Table 1 |
| Risk of bias in studies | 18 | Present assessments of risk of bias for each included study. | Table S8, Table S9 |
| Results of individual studies | 19 | For all outcomes, present, for each study: (a) summary statistics for each group (where appropriate) and (b) an effect estimate and its precision (e.g. confidence/credible interval), ideally using structured tables or plots. | Table 2, Table 3 |
| Results of syntheses | 20a | For each synthesis, briefly summarise the characteristics and risk of bias among contributing studies. | Results |
|  | 20b | Present results of all statistical syntheses conducted. If meta-analysis was done, present for each the summary estimate and its precision (e.g. confidence/credible interval) and measures of statistical heterogeneity. If comparing groups, describe the direction of the effect. | Figure 2, Figure 3 |
|  | 20c | Present results of all investigations of possible causes of heterogeneity among study results. | Table 2, Table 3 |
|  | 20d | Present results of all sensitivity analyses conducted to assess the robustness of the synthesized results. | Results |
| Reporting biases | 21 | Present assessments of risk of bias due to missing results (arising from reporting biases) for each synthesis assessed. | Results |
| Certainty of evidence | 22 | Present assessments of certainty (or confidence) in the body of evidence for each outcome assessed. | Results |
| **DISCUSSION** | | |  |
| Discussion | 23a | Provide a general interpretation of the results in the context of other evidence. | Discussion |
|  | 23b | Discuss any limitations of the evidence included in the review. | Discussion |
|  | 23c | Discuss any limitations of the review processes used. | Discussion |
|  | 23d | Discuss implications of the results for practice, policy, and future research. | Discussion |
| **OTHER INFORMATION** | | |  |
| Registration and protocol | 24a | Provide registration information for the review, including register name and registration number, or state that the review was not registered. | Methods |
|  | 24b | Indicate where the review protocol can be accessed, or state that a protocol was not prepared. | Methods |
|  | 24c | Describe and explain any amendments to information provided at registration or in the protocol. | Methods |
| Support | 25 | Describe sources of financial or non-financial support for the review, and the role of the funders or sponsors in the review. | Declaration |
| Competing interests | 26 | Declare any competing interests of review authors. | Declaration |
| Availability of data, code and other materials | 27 | Report which of the following are publicly available and where they can be found: template data collection forms; data extracted from included studies; data used for all analyses; analytic code; any other materials used in the review. | Table S6, Table S7 |

**Table S2.**

Full search strategy in electronic databases.

| APA PsycINFO | Results |
| --- | --- |
| 1. Anywhere=(diabetes mellitus) | **18,985** |
| 2. Anywhere=(depression) | **419,213** |
| 3. Anywhere=(depressive disorder OR depressive) | **176,789** |
| 4. Anywhere=(depress*) | **465,879** |
| 5. Anywhere=(major depression) | **188,306** |
| 6. 2 OR 3 OR 4 OR 5 | **465,879** |
| 7. Anywhere=(mortality) | **56,687** |
| 8. Anywhere=(prognosis) | **37,489** |
| 9. 7 OR 8 | **91,556** |
| 10. 1 AND 6 AND 9 | **275** |
| 11. Anywhere=(complications) | **39,968** |
| 12. Anywhere=(microvascular) | **1,692** |
| 13. Anywhere=(macrovascular) | **160** |
| 14. Anywhere=(retinopathy OR maculopathy) | **1,238** |
| 15. Anywhere=(nephropathy OR kidney disease) | **6,388** |
| 16. Anywhere=(neuropathy) | **9,689** |
| 17. Anywhere=(peripheral arterial disease OR diabetic foot OR amputation) | **3,035** |
| 18. Anywhere=(atherosclerosis OR myocardial infarction OR cardiovascular OR heart failure OR arrhythmia) | **77,281** |
| 19. Anywhere=(stroke OR cerebrovascular) | **61,857** |
| 20. Anywhere=(ketoacidosis OR coma OR hyperglycemia) | **13,293** |
| 21. 11 OR 12 OR 13 OR 14 OR 15 OR 16 OR 17 OR 18 OR 19 OR 20 | **185,456** |
| 22. 1 AND 6 AND 21 | **1,439** |
| 23. 11 OR 22 | **1,529** |
| EMBASE | Results |
| 1. ALL=(diabetes mellitus.mp. OR exp diabetes mellitus/) | 1,402,805 |
| 2. ALL=(depression.mp.) | 934,801 |
| 3. ALL=(depressive disorder.mp OR exp depression/) | 694,375 |
| 4. ALL=(depress*.mp.) | 1,059,086 |
| 5. ALL=(major depression.mp.) | 105,305 |
| 6. 2 OR 3 OR 4 OR 5 | 1,106,398 |
| 7. ALL=(mortality.mp. OR exp mortality/) | 2,101,177 |
| 8. ALL=(exp prognosis/ OR prognosis.mp.) | 1,327,677 |
| 9. 7 OR 8 | 3,184,476 |
| 10. 1 AND 6 AND 9 | 8,485 |
| 11. ALL=(complications.mp. OR exp complication/) | 3,207,143 |
| 12. ALL=(microvascular.mp.) | 118,841 |
| 13. ALL=(macrovascular.mp.) | 13,355 |
| 14. 11 OR 12 OR 13 | 3,303,842 |
| 15. ALL=(exp retinopathy/ OR retinopathy.mp.) | 73,590 |
| 16. ALL=(maculopathy.mp. OR exp retina maculopathy/) | 151,015 |
| 17. 15 OR 16 | 206,116 |
| 18. ALL=(nephropathy.mp. OR exp kidney disease/) | 1,277,668 |
| 19. ALL=(neuropathy.mp. OR exp neuropathy/) | 1,008,639 |
| 20. ALL=(peripheral arterial disease.mp. OR exp peripheral arterial disease/) | 49,528 |
| 21. ALL=(diabetic foot.mp. OR exp diabetic foot/) | 26,739 |
| 22. ALL=(exp amputation/ OR amputation.mp.) | 82,523 |
| 23. 20 OR 21 OR 22 | 141,388 |
| 24. ALL=(exp atherosclerosis/ OR atherosclerosis.mp.) | 369,706 |
| 25. ALL=(myocardial infarction.mp. OR exp heart infarction/) | 529,050 |
| 26. ALL=(cardiovascular.mp.) | 1,380,603 |
| 27. ALL=(heart failure.mp. OR exp heart failure/) | 770,611 |
| 28. ALL=(arrhythmia.mp. OR exp heart arrhythmia/) | 674,198 |
| 29. 24 OR 25 OR 26 OR 27 OR 28 | 2,810,803 |
| 30. ALL=(stroke.mp. OR exp cerebrovascular accident/) | 701,365 |
| 31. ALL=(cerebrovascular.mp.) | 455,140 |
| 32. 30 OR 31 | 797,513 |
| 33. ALL=(exp ketoacidosis/ OR ketoacidosis.mp.) | 32,938 |
| 34. ALL=(coma.mp. OR exp coma/) | 123,516 |
| 35. ALL=(hyperglycemia.mp. OR exp hyperglycemia/) | 156,396 |
| 36. 33 OR 34 OR 35 | 301,275 |
| 37. 14 OR 17 OR 18 OR 19 OR 23 OR 29 OR 32 OR 36 | 7,929,532 |
| 38. 1 AND 6 AND 37 | 35,660 |
| 39. 10 OR 38 | 37,030 |
| MEDLINE | Results |
| 1. ALL=(diabetes mellitus) | 645,596 |
| 2. ALL=(depression) | 667,167 |
| 3. ALL=(depressive disorder OR depressive) | 667,167 |
| 4. ALL=(depress*) | 704,225 |
| 5. ALL=(major depression) | 191,964 |
| 6. 2 OR 3 OR 4 OR 5 | 705,041 |
| 7. ALL=(mortality) | 1,649,239 |
| 8. ALL=(prognosis) | 2,377,788 |
| 9. 7 OR 8 | 3,513,543 |
| 10. 1 AND 6 AND 9 | 2,220 |
| 11. ALL=(complications) | 4,037,603 |
| 12. ALL=(microvascular) | 81,666 |
| 13. ALL=(macrovascular) | 8,729 |
| 14. ALL=(retinopathy OR maculopathy) | 210,568 |
| 15. ALL=(nephropathy OR kidney disease) | 793,173 |
| 16. ALL=(neuropathy) | 114,687 |
| 17. ALL=(peripheral arterial disease OR diabetic foot OR amputation) | 114,242 |
| 18. ALL=(atherosclerosis OR myocardial infarction OR cardiovascular OR heart failure OR arrhythmia) | 2,778,974 |
| 19. ALL=(stroke OR cerebrovascular) | 591,581 |
| 20. ALL=(ketoacidosis OR coma OR hyperglycemia) | 168,418 |
| 21. 11 OR 12 OR 13 OR 14 OR 15 OR 16 OR 17 OR 18 OR 19 OR 20 | 7,219,022 |
| 22. 1 AND 6 AND 21 | 8,658 |
| 23. 10 OR 22 | 9,193 |
| Web of Science | Results |
| 1. Topic=(diabetes mellitus) | 383,294 |
| 2. Topic=(depression) | 734,886 |
| 3. Topic=(depressive disorder OR depressive) | 218,736 |
| 4. Topic=(depress*) | 928,483 |
| 5. Topic=(major depression) | 119,504 |
| 6. 2 OR 3 OR 4 OR 5 | 928,483 |
| 7. Topic=(mortality) | 1,496,269 |
| 8. Topic=(prognosis) | 670,338 |
| 9. 7 OR 8 | 2,065,092 |
| 10. 1 AND 6 AND 9 | 1,672 |
| 11. Topic=(complications) | 1,251,453 |
| 12. Topic=(microvascular) | 97,898 |
| 13. Topic=(macrovascular) | 8,956 |
| 14. Topic=(retinopathy OR maculopathy) | 87,603 |
| 15. Topic=(nephropathy OR kidney disease) | 358,297 |
| 16. Topic=(neuropathy) | 125,865 |
| 17. Topic=(peripheral arterial disease OR diabetic foot OR amputation) | 93,565 |
| 18. Topic=(atherosclerosis OR myocardial infarction OR cardiovascular OR heart failure OR arrhythmia) | 1,521,235 |
| 19. Topic=(stroke OR cerebrovascular) | 551,970 |
| 20. Topic=(ketoacidosis OR coma OR hyperglycemia) | 135,756 |
| 21. 11 OR 12 OR 13 OR 14 OR 15 OR 16 OR 17 OR 18 OR 19 OR 20 | 3,667,929 |
| 22. 1 AND 6 AND 21 | 4,945 |
| 23. 10 OR 22 | 5,429 |

# List of included studies for mortality outcomes

1. **Ho MTH, Chan JKN, Lo HKY, Fang CZ, Wong CSM, Lee KCK, Lai FTT, Ng APP, Chen KQ, Wong WCW and Chang WC** (2025) Risk of mortality and complications in people with depressive disorder and diabetes mellitus: A 20-year population-based propensity score-matched cohort study. *European Neuropsychopharmacology* **92**, 10-18.
2. **Li G, Yu Y, Lin C, Zheng S, Tu H and Xu W** (2025) Association between major depressive disorder or depressive symptoms and the risk of vascular complications among patients with type 2 diabetes, and the mediating role of metabolic biomarkers: An analysis of the UK Biobank cohort. *eClinicalMedicine* **79**, 102982.
3. **Kim S, Kim G, Cho SH, Oh R, Kim JY, Lee YB, Jin SM, Hur KY and Kim JH** (2024) Impact of mental disorders on the all-cause mortality and cardiovascular disease outcomes in adults with new-onset type 1 diabetes: A nationwide cohort study. *Psychiatry Research* **342**, 116228.
4. **Fleetwood KJ, Wild SH, Licence KAM, Mercer SW, Smith DJ and Jackson CA** (2023) Severe mental illness and type 2 diabetes outcomes and complications: a nationwide cohort study. *Diabetes Care* **46**(7), 1363-1371.
5. **Kim H, Lee KN, Shin DW, Han K and Jeon HJ** (2022) Association of comorbid mental disorders with cardiovascular disease risk in patients with type 2 diabetes: A nationwide cohort study. *General Hospital Psychiatry* **79**, 33-41.
6. **Lunghi C, Zongo A, Tardif I, Demers É, Diendéré JDR and Guénette L** (2021) Depression but not non-persistence to antidiabetic drugs is associated with mortality in type 2 diabetes: A nested case-control study. *Diabetes Research and Clinical Practice* **171**, 108566.
7. **Jung I, Kwon H, Park SE, Han KD, Park YG, Kim YH, Rhee EJ and Lee WY** (2021) Increased risk of cardiovascular disease and mortality in patients with diabetes and coexisting depression: A nationwide population-based cohort study. *Diabetes and Metabolism Journal* **45**(3), 379-389.
8. **Wu CS, Hsu LY and Wang SH** (2020) Association of depression and diabetes complications and mortality: A population-based cohort study. *Epidemiology and Psychiatric Sciences* **29**, e96.
9. **Fernández de Alba I, Gimeno-Miguel A, Poblador-Plou B, Gimeno-Feliu LA, Ioakeim-Skoufa I, Rojo-Martínez G, Forjaz MJ and Prados-Torres A** (2020) Association between mental health comorbidity and health outcomes in type 2 diabetes mellitus patients. *Scientific Reports* **10**(1), 19583.
10. **Chiang JI, Hanlon P, Li T-C, Jani BD, Manski-Nankervis J-A, Furler J, Lin C-C, Yang S-Y, Nicholl BI, Thuraisingam S and Mair FS** (2020) Multimorbidity, mortality, and HbA1c in type 2 diabetes: A cohort study with UK and Taiwanese cohorts. *PLOS Medicine* **17**(5), e1003094.
11. **Jeong JH, Um YH, Ko SH, Park JH, Park JY, Han K and Ko KS** (2017) Depression and mortality in people with type 2 diabetes mellitus, 2003 to 2013: A nationwide population-based cohort study. *Diabetes and Metabolism Journal* **41**(4), 296-302.
12. **Novak M, Mucsi I, Rhee CM, Streja E, Lu JL, Kalantar-Zadeh K, Molnar MZ and Kovesdy CP** (2016) Increased risk of incident chronic kidney disease, cardiovascular disease, and mortality in patients with diabetes with comorbid depression. *Diabetes Care* **39**(11), 1940-1947.
13. **Lynch CP, Gebregziabher M, Zhao Y, Hunt KJ and Egede LE** (2014) Impact of medical and psychiatric multi-morbidity on mortality in diabetes: Emerging evidence. *BMC Endocrine Disorders* **14**, 68.
14. **Ting RZ, Lau ES, Ozaki R, Lau WW, Kong AP, Luk AO, Chow CC, Ma RC, Wing YK, Chan JC and So WY** (2013) High risk for cardiovascular disease in Chinese type 2 diabetic patients with major depression - A 7-year prospective analysis of the Hong Kong Diabetes Registry. *Journal of Affective Disorders* **149**(1-3), 129-135
15. **Winkley K, Sallis H, Kariyawasam D, Leelarathna LH, Chalder T, Edmonds ME, Stahl D and Ismail K** (2012) Five-year follow-up of a cohort of people with their first diabetic foot ulcer: the persistent effect of depression on mortality. *Diabetologia* **55**(2), 303-310.
16. **Mai Q, Holman CD, Sanfilippo FM, Emery JD and Preen DB** (2011) Mental illness related disparities in diabetes prevalence, quality of care and outcomes: A population-based longitudinal study. *BMC Medicine* **9**, 118.
17. **Katon W, Fan MY, Unützer J, Taylor J, Pincus H and Schoenbaum M** (2008) Depression and diabetes: A potentially lethal combination. *Journal of General Internal Medicine* **23**(10), 1571-1575.
18. **Richardson LK, Egede LE and Mueller M** (2008) Effect of race/ethnicity and persistent recognition of depression on mortality in elderly men with type 2 diabetes and depression. *Diabetes Care* **31**(5), 880-881.

# List of included studies for complication outcomes

1. **Ho MTH, Chan JKN, Lo HKY, Fang CZ, Wong CSM, Lee KCK, Lai FTT, Ng APP, Chen KQ, Wong WCW and Chang WC** (2025) Risk of mortality and complications in people with depressive disorder and diabetes mellitus: A 20-year population-based propensity score-matched cohort study. *European Neuropsychopharmacology* **92**, 10-18.
2. **Li G, Yu Y, Lin C, Zheng S, Tu H and Xu W** (2025) Association between major depressive disorder or depressive symptoms and the risk of vascular complications among patients with type 2 diabetes, and the mediating role of metabolic biomarkers: an analysis of the UK Biobank cohort. *eClinicalMedicine* **79**, 102982.
3. **Kim S, Kim G, Cho SH, Oh R, Kim JY, Lee YB, Jin SM, Hur KY and Kim JH** (2024) Impact of mental disorders on the all-cause mortality and cardiovascular disease outcomes in adults with new-onset type 1 diabetes: a nationwide cohort study. *Psychiatry Research* **342**, 116228.
4. **Scheuer SH, Andersen GS, Carstensen B, Diaz L, Kosjerina V, Lindekilde N, Wild SH, Jackson CA, Pouwer F, Benros ME and Jørgensen ME** (2024) Trends in incidence of hospitalization for hypoglycemia and diabetic ketoacidosis in individuals with type 1 or type 2 diabetes with and without severe mental illness in Denmark from 1996 to 2020: A nationwide study. *Diabetes Care* **47**(6), 1065-1073.
5. **Yoo TK, Han KD, Rhee EJ and Lee WY** (2023) Impact of mental disorders on the risk of heart failure among Korean patients with diabetes: A cohort study. *Cardiovascular Diabetology* **22**(1), 115.
6. **Fleetwood KJ, Wild SH, Licence KAM, Mercer SW, Smith DJ and Jackson CA** (2023) Severe mental illness and type 2 diabetes outcomes and complications: A nationwide cohort study. *Diabetes Care* **46**(7), 1363-1371.
7. **Bae NY, Lee SR, Choi EK, Ahn HJ, Ahn HJ, Kwon S, Han KD, Lee KN, Oh S and Lip GYH** (2022) Impact of mental disorders on the risk of atrial fibrillation in patients with diabetes mellitus: A nationwide population-based study. *Cardiovascular Diabetology* **21(1)**, 251.
8. **Kim H, Lee KN, Shin DW, Han K and Jeon HJ** (2022) Association of comorbid mental disorders with cardiovascular disease risk in patients with type 2 diabetes: A nationwide cohort study. *General Hospital Psychiatry* **79**, 33-41.
9. **Scheuer SH, Kosjerina V, Lindekilde N, Pouwer F, Carstensen B, Jørgensen ME, Benros ME and Andersen GS** (2022) Severe mental illness and the risk of diabetes complications: A nationwide, register-based cohort study. *Journal of Clinical Endocrinology and Metabolism* **107**(8), e3504-e3514.
10. **Jung I, Kwon H, Park SE, Han KD, Park YG, Kim YH, Rhee EJ and Lee WY** (2021) Increased risk of cardiovascular disease and mortality in patients with diabetes and coexisting depression: A nationwide population-based cohort study. *Diabetes and Metabolism Journal* **45**(3), 379-389.
11. **Wu CS, Hsu LY and Wang SH** (2020) Association of depression and diabetes complications and mortality: A population-based cohort study. *Epidemiology and Psychiatric Sciences* **29**, e96.
12. **Lee HM, Yang YC, Chen SF, Hsu CY and Shen YC** (2020) Risk of hyperglycemic crisis episode in diabetic patients with depression: A nationwide population-based cohort study. *Journal of Diabetes and its Complications* **34**(3), 107509.
13. **Novak M, Mucsi I, Rhee CM, Streja E, Lu JL, Kalantar-Zadeh K, Molnar MZ and Kovesdy CP** (2016) Increased risk of incident chronic kidney disease, cardiovascular disease, and mortality in patients with diabetes with comorbid depression. *Diabetes Care* **39**(11), 1940-1947.
14. **Ting RZ, Lau ES, Ozaki R, Lau WW, Kong AP, Luk AO, Chow CC, Ma RC, Wing YK, Chan JC and So WY** (2013) High risk for cardiovascular disease in Chinese type 2 diabetic patients with major depression - A 7-year prospective analysis of the Hong Kong Diabetes Registry. *Journal of Affective Disorders* **149**(1-3), 129-135.
15. **Williams LH, Miller DR, Fincke G, Lafrance JP, Etzioni R, Maynard C, Raugi GJ and Reiber GE** (2011) Depression and incident lower limb amputations in veterans with diabetes. *Journal of Diabetes and its Complications* **25**(3), 175-182.
16. **Clouse RE, Lustman PJ, Freedland KE, Griffith LS, McGill JB and Carney RM** (2003) Depression and coronary heart disease in women with diabetes. *Psychosomatic Medicine* **65**(3), 376-383.

**Table S3.**

List of excluded studies and the reasons for exclusions.

| Citations | Reasons for exclusion |
| --- | --- |
| Yeung et al. (2024) | No ICD/DSM criteria for depressive disorder |
| Xu et al. (2024) | No ICD/DSM criteria for depressive disorder |
| Tu et al. (2024) | No ICD/DSM criteria for depressive disorder |
| Rivera-Santiago et al. (2024) | No relevant outcome estimates |
| Li et al. (2024) | No ICD/DSM criteria for depressive disorder |
| Wang et al. (2023) | No relevant outcome estimates |
| Lyu et al. (2023) | No ICD/DSM criteria for depressive disorder |
| Jiang et al. (2023) | No ICD/DSM criteria for depressive disorder |
| Feng et al. (2023) | No ICD/DSM criteria for depressive disorder |
| Tardif et al. (2022) | No relevant outcome estimates |
| Rohde et al. (2022) | No ICD/DSM criteria for depressive disorder |
| Prigge et al. (2022) | No relevant comparison group |
| Chen et al. (2022) | No ICD/DSM criteria for depressive disorder |
| Salinero-Fort et al. (2021) | No ICD/DSM criteria for depressive disorder |
| Leutner et al. (2021) | No relevant outcome estimates |
| Han et al. (2021) | No exposure in depressive disorder |
| Cole et al. (2021) | No exposure in depressive disorder |
| Hayashino et al. (2020) | No ICD/DSM criteria for depressive disorder |
| Mansori et al. (2019) | No ICD/DSM criteria for depressive disorder |
| Goins et al. (2019) | No ICD/DSM criteria for depressive disorder |
| Fung et al. (2018) | No ICD/DSM criteria for depressive disorder |
| Pan et al. (2017) | No ICD/DSM criteria for depressive disorder |
| Naicker et al. (2017) | No ICD/DSM criteria for depressive disorder |
| Ismail et al. (2017) | No ICD/DSM criteria for depressive disorder |
| Cummings et al. (2016) | No ICD/DSM criteria for depressive disorder |
| Bruce et al. (2016) | No ICD/DSM criteria for depressive disorder |
| Zhang et al. (2015) | No ICD/DSM criteria for depressive disorder |
| Reeves et al. (2015) | No ICD/DSM criteria for depressive disorder |
| Kikuchi et al. (2015) | No ICD/DSM criteria for depressive disorder |
| Iversen et al. (2015) | No ICD/DSM criteria for depressive disorder |
| Ribe et al. (2014) | No relevant comparison group |
| Yu et al. (2013) | No ICD/DSM criteria for depressive disorder |
| Loprinzi et al. (2013) | No ICD/DSM criteria for depressive disorder |
| Katon et al. (2013) | No ICD/DSM criteria for depressive disorder |
| Coleman et al. (2013) | No ICD/DSM criteria for depressive disorder |
| Sullivan et al. (2012) | No ICD/DSM criteria for depressive disorder |
| Green et al. (2012) | No ICD/DSM criteria for depressive disorder |
| Sieu et al. (2011) | No ICD/DSM criteria for depressive disorder |
| Scherrer et al. (2011) | No relevant comparison group |
| Prisciandaro et al. (2011) | No exposure in depressive disorder |
| Pan et al. (2011) | No ICD/DSM criteria for depressive disorder |
| Leung at el. (2011) | No exposure in depressive disorder |
| Le et al. (2011) | No relevant outcome estimates |
| Honkasalo et al. (2011) | No ICD/DSM criteria for depressive disorder |
| Davydow et al. (2011) | No ICD/DSM criteria for depressive disorder |
| Lin et al. (2010) | No ICD/DSM criteria for depressive disorder |
| Lin et al. (2009) | No ICD/DSM criteria for depressive disorder |
| Spitzer et al. (2008) | No ICD/DSM criteria for depressive disorder |
| Winkley et al. (2007) | Data overlapped with Winkley et al. 2012 (included study) |
| Tseng et al. (2007) | No exposure in depressive disorder |
| Moreira et al. (2007) | No adequate sample size |
| Katon et al. (2005) | No ICD/DSM criteria for depressive disorder |
| Bruce et al. (2005) | No ICD/DSM criteria for depressive disorder |
| Lin et al. (2004) | No relevant outcome estimates |
| Katon et al. (2004) | No ICD/DSM criteria for depressive disorder |
| Black et al. (2003) | No relevant comparison group |

# References

1. **Yeung YL, Lee KL, Lau ES, Yung TF, Yang A, Wu H, Wong KT, Kong AP, Chow EY, Ma RC, Yeung T, Loo K-m, Ozaki R, Luk AO, Lui JN and Chan JC** (2024) Associations of comorbid depression with cardiovascular-renal events and all-cause mortality accounting for patient reported outcomes in individuals with type 2 diabetes: A 6-year prospective analysis of the Hong Kong Diabetes Register. *Frontiers in Endocrinology* **15**, 1284799.
2. **Xu X, Feng C, Han H, Li T, Wang N, Yang Q, Guo Y, Gan X, Liu X, Sun L, Dregan A, Zong G and Gao X** (2024) Prospective study of depressive symptoms and incident cardiovascular diseases in people with type 2 diabetes. *Journal of Affective Disorders* **345**, 427-434.
3. **Tu Q, Hyun K, Lin S, Hafiz N, Manandi D, Zhang Q, Wang X, Zhang N, Wu H and Redfern J** (2024) Individual and joint effects of diabetes and depression on incident cardiovascular diseases and all-cause mortality: Results from a population-based cohort study. *Journal of Diabetes and its Complications* **38**(11), 108878.
4. **Rivera-Santiago K, Cumba-Aviles E and Gómez-Rivera D** (2024) Recurrent depression relates to worse outcomes than single episode depression among Hispanic adolescents with diabetes. *Health Psychology Report* **12**(1), 1-13.
5. **Li YH, Cheng YC, Liu HC, Wu J and Lee IT** (2024) Depressive symptoms associated with peripheral artery disease and predicting mortality in Type 2 diabetes. *Biomedicines* **12**(1), 29.
6. **Wang SH, Hsu LY, Lin MC and Wu CS** (2023) Associations between depression and cancer risk among patients with diabetes mellitus: A population-based cohort study. *Cancer Medicine* **12**(19), 19968-19977.
7. **Lyu Z, Chen Y, Zhu Z, Luo X, Cui Y, Xie J, Chen Z, Liu J, Wu X, Bulloch G and Meng Q** (2023) Associations of concomitant retinopathy and depression with mortality in a nationally representative population. *Journal of Affective Disorders* **336**, 15-24.
8. **Jiang G, Wang Y, Wang L, Chen M and Li W** (2023) The mediating effect of depression on new-onset stroke in diabetic population: evidence from the China health and retirement longitudinal study. *Journal of Affective Disorders* **321**, 208-216.
9. **Feng Z, Tong WK, Zhang X and Tang Z** (2023) Prevalence of depression and association with all-cause and cardiovascular mortality among individuals with type 2 diabetes: a cohort study based on NHANES 2005–2018 data. *BMC Psychiatry* **23**(1), 490.
10. **Tardif I, Guénette L, Zongo A, Demers É and Lunghi C** (2022) Depression and the risk of hospitalization in type 2 diabetes patients: A nested case-control study accounting for non-persistence to antidiabetic treatment. *Diabetes and Metabolism* **48**(4), 101334.
11. **Rohde C, Nielsen JS, Schöllhammer Knudsen J, Thomsen RW and Østergaard SD** (2022) Risk factors associated with mortality among individuals with type 2 diabetes and depression across two cohorts. *European Journal of Endocrinology* **187**(4), 567-577.
12. **Prigge R, Wild SH and Jackson CA** (2022) Depression, diabetes, comorbid depression and diabetes and risk of all-cause and cause-specific mortality: A prospective cohort study. *Diabetologia* **65**(9), 1450-1460.
13. **Chen VC, Wang TN, Hsieh MC, Chou SY, Lee MC, McIntyre RS, Lu ML, Liao YT and Yeh CJ** (2022) Depression, diabetes mellitus and mortality in older adults: A national cohort study in Taiwan. *Neuropsychiatric Disease and Treatment* **18**, 2639-2648.
14. **Salinero-Fort MA, Gómez-Campelo P, Cárdenas-Valladolid J, San Andrés-Rebollo FJ, de Miguel-Yanes JM and de Burgos-Lunar C** (2021) Effect of depression on mortality in type 2 diabetes mellitus after 8 years of follow-up. The DIADEMA study. *Diabetes Research and Clinical Practice* **176**, 108863.
15. **Leutner M, Haug N, Bellach L, Dervic E, Kautzky A, Klimek P and Kautzky-Willer A** (2021) Risk of typical diabetes-associated complications in different clusters of diabetic patients: Analysis of nine risk factors. *Journal of Personalized Medicine* **11**(5).
16. **Han L, Doran T, Holt RIG, Hewitt C, Jacobs R, Prady SL, Alderson SL, Shiers D, Wang HI, Bellass S, Gilbody S, Kitchen CEW, Lister J, Taylor J and Siddiqi N** (2021) Impact of severe mental illness on healthcare use and health outcomes for people with type 2 diabetes: A longitudinal observational study in England. *British Journal of General Practice* **71**(709), e565-e573.
17. **Coles B, Zaccardi F, Hvid C, Davies MJ and Khunti K** (2021) Cardiovascular events and mortality in people with type 2 diabetes and multimorbidity: A real-world study of patients followed for up to 19 years. *Diabetes, Obesity and Metabolism* **23**(1), 218-227.
18. **Hayashino Y, Okamura S, Tsujii S and Ishii H** (2020) The joint association of diabetes distress and depressive symptoms with all-cause mortality in Japanese individuals with type 2 diabetes: A prospective cohort study (Diabetes Distress and Care Registry in Tenri [DDCRT 20]). *Diabetologia* **63**(12), 2595-2604.
19. **Mansori K, Shiravand N, Shadmani FK, Moradi Y, Allahmoradi M, Ranjbaran M, Ahmadi S, Farahani A, Samii K and Valipour M** (2019) Association between depression with glycemic control and its complications in type 2 diabetes. *Diabetology & Metabolic Syndrome* **13**(2), 1555-1560.
20. **Goins RT, Noonan C, Winchester B and Brock D** (2019) Depressive symptoms and all‐cause mortality in older American Indians with type 2 diabetes mellitus. *Journal of the American Geriatrics Society* **67**(9), 1940-1945.
21. **Fung ACH, Tse G, Cheng HL, Lau ESH, Luk A, Ozaki R, So TTY, Wong RYM, Tsoh J, Chow E, Wing YK, Chan JCN and Kong APS** (2018) Depressive symptoms, co-morbidities, and glycemic control in Hong Kong Chinese elderly patients with type 2 diabetes mellitus. *Frontiers in Endocrinology* **9**, 261.
22. **Pan S, Liu ZW, Shi S, Ma X, Song WQ, Guan GC, Zhang Y, Zhu SM, Liu FQ, Liu B, Tang ZG, Wang JK and Lv Y** (2017) Hamilton rating scale for depression-24 (HAM-D24) as a novel predictor for diabetic microvascular complications in type 2 diabetes mellitus patients. *Psychiatry Research* **258**, 177-183.
23. **Naicker K, Johnson JA, Skogen JC, Manuel D, Øverland S, Sivertsen B and Colman I** (2017) Type 2 diabetes and comorbid symptoms of depression and anxiety: Longitudinal associations with mortality risk. *Diabetes Care* **40**(3), 352-358.
24. **Ismail K, Moulton CD, Winkley K, Pickup JC, Thomas SM, Sherwood RA, Stahl D and Amiel SA** (2017) The association of depressive symptoms and diabetes distress with glycaemic control and diabetes complications over 2 years in newly diagnosed type 2 diabetes: A prospective cohort study. *Diabetologia* **60**(10), 2092-2102.
25. **Cummings DM, Kirian K, Howard G, Howard V, Yuan Y, Muntner P, Kissela B, Redmond N, Judd SE and Safford MM** (2016) Consequences of comorbidity of elevated stress and/or depressive symptoms and incident cardiovascular outcomes in diabetes: Results from the REasons for Geographic And Racial Differences in Stroke (REGARDS) Study. *Diabetes Care* **39**(1), 101-109
26. **Bruce DG, Davis WA, Dragovic M, Davis TM and Starkstein SE** (2016) Comorbid anxiety and depression and their impact on cardiovascular disease in type 2 diabetes: The Fremantle Diabetes Study Phase II. *Depression and Anxiety* **33**(10), 960-966.
27. **Zhang Y, Ting RZ, Yang W, Jia W, Li W, Ji L, Guo X, Kong AP, Wing YK, Luk AO, Sartorius N, Morisky DE, Oldenburg B, Weng J and Chan JC** (2015) Depression in Chinese patients with type 2 diabetes: Associations with hyperglycemia, hypoglycemia, and poor treatment adherence. *Journal of Diabetes* **7**(6), 800-808.
28. **Reeves GC, Alhurani AS, Frazier SK, Watkins JF, Lennie TA and Moser DK** (2015) The association of comorbid diabetes mellitus and symptoms of depression with all-cause mortality and cardiac rehospitalization in patients with heart failure. *BMJ Open Diabetes and Research Care* **3**(1), e000077.
29. **Kikuchi Y, Iwase M, Fujii H, Ohkuma T, Kaizu S, Ide H, Jodai T, Idewaki Y, Nakamura U and Kitazono T** (2015) Association of severe hypoglycemia with depressive symptoms in patients with type 2 diabetes: the Fukuoka Diabetes Registry. *BMJ Open Diabetes and Research Care* **3**(1), e000063.
30. **Iversen MM, Tell GS, Espehaug B, Midthjell K, Graue M, Rokne B, Berge LI and Østbye T** (2015) Is depression a risk factor for diabetic foot ulcers? 11-years follow-up of the Nord-Trøndelag Health Study (HUNT). *Journal of Diabetes and its Complications* **29**(1), 20-25.
31. **Ribe AR, Laursen TM, Sandbaek A, Charles M, Nordentoft M and Vestergaard M** (2014) Long-term mortality of persons with severe mental illness and diabetes: A population-based cohort study in Denmark. *Psychological Medicine* **44**(14), 3097-3107.
32. **Yu MK, Katon W and Young BA** (2013) Diabetes self-care, major depression, and chronic kidney disease in an outpatient diabetic population. *Nephron Clinical Practice* **124**(1-2), 106-112.
33. **Loprinzi PD, Smit E and Pariser G** (2013) Association among depression, physical functioning, and hearing and vision impairment in adults with diabetes. *Diabetes Spectrum* **26**(1), 6-15.
34. **Katon WJ, Young BA, Russo J, Lin EH, Ciechanowski P, Ludman EJ and Von Korff MR** (2013) Association of depression with increased risk of severe hypoglycemic episodes in patients with diabetes. *Annals of Family Medicine* **11**(3), 245-250.
35. **Coleman SM, Katon W, Lin E and Von Korff M** (2013) Depression and death in diabetes; 10-year follow-up of all-cause and cause-specific mortality in a diabetic cohort. *Psychosomatics* **54**(5), 428-436.
36. **Sullivan MD, O'Connor P, Feeney P, Hire D, Simmons DL, Raisch DW, Fine LJ, Narayan KM, Ali MK and Katon WJ** (2012) Depression predicts all-cause mortality: epidemiological evaluation from the ACCORD HRQL substudy. *Diabetes Care* **35**(8), 1708-1715.
37. **Green AJ, Fox KM and Grandy S** (2012) Self-reported hypoglycemia and impact on quality of life and depression among adults with type 2 diabetes mellitus. *Diabetes Research and Clinical Practice* **96**(3), 313-318.
38. **Sieu N, Katon W, Lin EH, Russo J, Ludman E and Ciechanowski P** (2011) Depression and incident diabetic retinopathy: A prospective cohort study. *General Hospital Psychiatry* **33**(5), 429-435.
39. **Scherrer JF, Garfield LD, Chrusciel T, Hauptman PJ, Carney RM, Freedland KE, Owen R, True WR and Lustman PJ** (2011) Increased risk of myocardial infarction in depressed patients with type 2 diabetes. *Diabetes Care* **34**(8), 1729-1734.
40. **Prisciandaro JJ, Gebregziabher M, Grubaugh AL, Gilbert GE, Echols C and Egede LE** (2011) Impact of psychiatric comorbidity on mortality in veterans with type 2 diabetes. *Diabetes Technology & Therapeutics* **13**(1), 73-78.
41. **Pan A, Lucas M, Sun Q, van Dam RM, Franco OH, Willett WC, Manson JE, Rexrode KM, Ascherio A and Hu FB** (2011) Increased mortality risk in women with depression and diabetes mellitus. *Archives of General Psychiatry* **68**(1), 42-50.
42. **Leung G, Zhang J, Lin WC and Clark RE** (2011) Behavioral disorders and diabetes-related outcomes among Massachusetts Medicare and Medicaid beneficiaries. *Psychiatric Services* **62**(6), 659-665.
43. **Le TK, Curtis B, Kahle-Wrobleski K, Johnston J, Haldane D and Melfi C** (2011) Treatment patterns and resource use among patients with comorbid diabetes mellitus and major depressive disorder. *Journal of Medical Economics* **14**(4), 440-447.
44. **Honkasalo MT, Elonheimo OM and Sane T** (2011) Severe hypoglycaemia in drug-treated diabetic patients needs attention: A population-based study. *Scandinavian Journal of Primary Health Care* **29**(3), 165-170.
45. **Davydow DS, Russo JE, Ludman E, Ciechanowski P, Lin EH, Von Korff M, Oliver M and Katon WJ** (2011) The association of comorbid depression with intensive care unit admission in patients with diabetes: A prospective cohort study. *Psychosomatics* **52**(2), 117-126.
46. **Lin EH, Rutter CM, Katon W, Heckbert SR, Ciechanowski P, Oliver MM, Ludman EJ, Young BA, Williams LH, McCulloch DK and Von Korff M** (2010) Depression and advanced complications of diabetes: A prospective cohort study. *Diabetes Care* **33**(2), 264-269.
47. **Lin EH, Heckbert SR, Rutter CM, Katon WJ, Ciechanowski P, Ludman EJ, Oliver M, Young BA, McCulloch DK and Von Korff M** (2009) Depression and increased mortality in diabetes: Unexpected causes of death. *Annals of Family Medicine* **7**(5), 414-421.
48. **Spitzer C, Völzke H, Barnow S, Krohn U, Wallaschofski H, Lüdemann J, John U, Freyberger HJ, Kerner W and Grabe HJ** (2008) Association between depression and subclinical carotid atherosclerosis in patients with type 1 diabetes. *Diabetic Medicine* **25**(3), 349-354.
49. **Winkley K, Stahl D, Chalder T, Edmonds ME and Ismail K** (2007) Risk factors associated with adverse outcomes in a population-based prospective cohort study of people with their first diabetic foot ulcer. *Journal of Diabetes and its Complications* **21**(6), 341-349.
50. **Tseng CL, Sambamoorthi U, Helmer D, Tiwari A, Rosen AK, Rajan M and Pogach L** (2007) The association between mental health functioning and nontraumatic lower extremity amputations in veterans with diabetes. *General Hospital Psychiatry* **29**(6), 537-546.
51. **Moreira RO, Papelbaum M, Fontenelle LF, Appolinario JC, Ellinger VC, Coutinho WF and Zagury L** (2007) Comorbidity of psychiatric disorders and symmetric distal polyneuropathy among type II diabetic outpatients. *Brazilian Journal of Medical and Biological Research* **40**(2), 269-275.
52. **Katon WJ, Rutter C, Simon G, Lin EH, Ludman E, Ciechanowski P, Kinder L, Young B and Von Korff M** (2005) The association of comorbid depression with mortality in patients with type 2 diabetes. *Diabetes Care* **28**(11), 2668-2672.
53. **Bruce DG, Davis WA, Starkstein SE and Davis TM** (2005) A prospective study of depression and mortality in patients with type 2 diabetes: The Fremantle Diabetes Study. *Diabetologia* **48**(12), 2532-2539.
54. **Lin EH, Katon W, Von Korff M, Rutter C, Simon GE, Oliver M, Ciechanowski P, Ludman EJ, Bush T and Young B** (2004) Relationship of depression and diabetes self-care, medication adherence, and preventive care. *Diabetes Care* **27**(9), 2154-2160.
55. **Katon WJ, Lin EH, Russo J, Von Korff M, Ciechanowski P, Simon G, Ludman E, Bush T and Young B** (2004) Cardiac risk factors in patients with diabetes mellitus and major depression. *Journal of General Internal Medicine* **19**(12), 1192-1199.
56. **Black SA, Markides KS and Ray LA** (2003) Depression predicts increased incidence of adverse health outcomes in older Mexican Americans with type 2 diabetes. *Diabetes Care* **26**(10), 2822-2828.

**Table S4.**

Additional characteristics of included studies.

| Author ^a^  (year) | Age criterion, years | Depression assessment  methods ^b^ | Depression diagnostic  system | Depression diagnostic  codes | DM assessment methods | Mortality/ Complication  assessment methods | Follow-up (years) |
| --- | --- | --- | --- | --- | --- | --- | --- |
| Ho  (2025) | ≥30 | Record linkage (psychiatric inpatient admission or outpatient attendance record in the Clinical Data Analysis and Reporting System) | ICD-10 | F32-F33, F34.1 | Record linkage (inpatient admission, specialist outpatient attendance or general outpatient attendance, or prescription record in the Clinical Data Analysis and Reporting System) | Record linkage (the Clinical Data Analysis and Reporting System) | 20 |
|  |  |  |  |  |  |  |  |
| Li  (2025) | 37-73 | Record linkage (hospital inpatient admission records from the UK Biobank) | ICD-10 | F32-F33 | Record linkage (the UK Biobank) | Record linkage (hospital inpatient admission, the vital statistics, the primary care data from the UK Biobank) | 16 |
|  |  |  |  |  |  |  |  |
| Kim  (2024) | ≥19 | Record linkage (the National Health Insurance System database) | ICD-10 | F32-F33 | Record linkage (the National Health Insurance System database) | Record linkage (the National Health Insurance System database) | 11 |
|  |  |  |  |  |  |  |  |
| Scheuer (2024) | ≥17 | Record linkage (the Danish Psychiatric Research Register) | ICD-10  ICD-8 | ICD-10: F32-F33  ICD-8: 296.09, 296.29, 298.09, 300.49 | Record linkage (the Danish Diabetes Registers) | Record linkage (the Danish National Patient Register) | 24 |
|  |  |  |  |  |  |  |  |
| Yoo  (2023) | ≥20 | Record linkage (the National Health Insurance Service records and the Korean National Health Screening data base) | ICD-10 | F32-F33 | Record linkage (the National Health Insurance Service records and the Korean National Health Screening data base) | Record linkage (the National Health Insurance Service records and the Korean National Health Screening data base) | 10 |
|  |  |  |  |  |  |  |  |
| Fleetwood  (2023) | ≥18 | Record linkage (the Scottish Diabetes Research Network National Diabetes Dataset, linked to acute and psychiatric hospital inpatient admission records) | ICD-10  ICD-9 | ICD-10: F32-F33  ICD-9: 296.1 298.0, 300.4, 311 | Record linkage (the Scottish Diabetes Research Network National Diabetes Dataset) | Record linkage (acute hospital and death records, the Scottish diabetic eye screening program, the Scottish Renal Registry, hospital records using the Office of Population Censuses and Surveys’ Classification of Surgical Operations and Procedures, 4th revision (OPCS-4) codes and death records) | 15 |
|  |  |  |  |  |  |  |  |
| Bae  (2022) | ≥20 | Record linkage (the National Health Information database) | ICD-10 | F32-F33 | Record linkage (the National Health Information database) | Record linkage (the National Health Information database) | 10 |
|  |  |  |  |  |  |  |  |
| Kim  (2022) | ≥20 | Record linkage (the National Health Insurance Sharing Service database) | ICD-10 | F32, F33, F34.1, F34.81 | Record linkage (the National Health Insurance Sharing Service database) | Record linkage (the National Health Insurance Sharing Service database) | 10 |
|  |  |  |  |  |  |  |  |
| Scheuer  (2022) | NA | Record linkage (the Danish Psychiatric Research Register) | ICD-10  ICD-8 | ICD-10: F32-F33  ICD-8: 296.09, 296.29, 298.09, 300.49 | Record linkage (the National Patient Register, the Danish National Health Service Register, the Danish National Prescription Registry, the Danish Adult Diabetes Registry and the Danish Registry of Diabetic Retinopathy) | Record linkage (the Danish National Patient Register, the Danish Register of Causes of Death and the Register of Laboratory Results) | 23 |
|  |  |  |  |  |  |  |  |
| Lunghi  (2021) | ≥18 | Record linkage (medico-administrative data from the Régie de l’assurance maladie du Québec) | ICD-10  ICD-9 | ICD-10: F32, F33, F34.1, F38.1  ICD-9: 296.2, 296.3, 300.4, 311 | Record linkage (medico-administrative data from the Régie de l’assurance maladie du Québec) | Record linkage (medico-administrative data from the Régie de l’assurance maladie du Québec) | 8 |
|  |  |  |  |  |  |  |  |
| Jung  (2021) | ≥30 | Record linkage (the Korean National Health Insurance Service (NHIS) and claims database) | ICD-10-CM | F32-F33 | Record linkage (the Korean National Health Insurance Service (NHIS) and claims database) | Record linkage (the Korean National Health Insurance Service (NHIS) and claims database) | 8 |
|  |  |  |  |  |  |  |  |
| Wu  (2020) | ≥20 | Record linkage (the National Health Insurance Research Database) | ICD-9-CM | 296.3x, 296.2x, 300.4, 311 | Record linkage (the National Health Insurance Research Database) | Record linkage (Taiwan’s National Death Registry) | 14 |
|  |  |  |  |  |  |  |  |
| Lee  (2020) | NA | Record linkage (the Longitudinal Cohort of Diabetes Patients from the National Health Insurance Programme claims data) | ICD-9-CM | 296.2, 296.3, 300.4, 311 | Record linkage (the Longitudinal Cohort of Diabetes Patients from the National Health Insurance Programme claims data) | Record linkage (the Longitudinal Cohort of Diabetes Patients from the National Health Insurance Programme claims data) | 14 |
|  |  |  |  |  |  |  |  |
| Fernández de Alba  (2020) | ≥18 | Record linkage (electronic health records and clinical-administrative databases linked to the EpiChron Cohort) | ICD-9-CM  ICPC-1 | PSY09 (grouped in the Expanded Diagnostic Clusters (EDCs) of the Johns Hopkins ACG System) | Record linkage (electronic health records and clinical-administrative databases linked to the EpiChron Cohort) | Record linkage (electronic health records and clinical-administrative databases linked to the EpiChron Cohort) | 4 |
|  |  |  |  |  |  |  |  |
| Chiang  (2020) | NA | Record linkage (Taiwan National Diabetes Care Management Program) | ICD-9 | NA | Record linkage (Taiwan National Diabetes Care Management Program) | Record linkage (Taiwan National Diabetes Care Management Program) | 10 |
|  |  |  |  |  |  |  |  |
|  | NA | Record linkage (UK Biobank) | ICD-10-CM | NA | Record linkage (UK Biobank) | Record linkage (UK Biobank) | 12 |
|  |  |  |  |  |  |  |  |
| Jeong  (2017) | ≥30 | Record linkage (the National Health Information databases by the Korean National Health Insurance Service [NHIS]) | ICD-10 | F320-F349 | Record linkage (the National Health Information databases by the Korean National Health Insurance Service [NHIS]) | Record linkage (Claims data and national mortality data from the National Statistical Office) | 10 |
|  |  |  |  |  |  |  |  |
| Novak  (2016) | NA | Record linkage (Veterans Affairs Inpatient and Out-patient Medical SAS Datasets) | ICD-9-CM | 296.x | Record linkage (Veterans Affairs Inpatient and Out-patient Medical SAS Datasets) | Record linkage (the Veterans Affairs Vital Status Files, Veterans Affairs Inpatient and Out-patient Medical SAS Datasets) | 9 |
|  |  |  |  |  |  |  |  |
| Lynch  (2014) | NA | Record linkage (The Veterans Health Administration National Patient Care database) | ICD-9 | NA | Record linkage (The Veterans Health Administration National Patient Care database and Pharmacy Benefits Management database) | Record linkage | 5 |
|  |  |  |  |  |  |  |  |
| Ting  (2013) | NA | Record linkage (Hospital Authority Central Computer System) | DSM-IV | NA | Record linkage (the Hong Kong Diabetes Registry) | Record linkage (Hospital Authority Central Computer System) | 13 |
|  |  |  |  |  |  |  |  |
| Winkley  (2012) | NA | Diagnostic research clinical interview by the WHO Schedules in Clinical Assessment and Neuropsychiatry (SCAN) 2.1 | DSM-IV | NA | Diagnostic interview and research record through contact with the clinics involved and review of previous records | Record linkage (UK’s Central Register Office) | 5 |
|  |  |  |  |  |  |  |  |
| Mai  (2011) | NA | Record linkage (the Western Australia state mental health registry) | ICD-9 | 311 | Record linkage (hospital discharge diagnoses, prescriptions from Pharmaceutical Benefits Scheme claims, Commonwealth Medicare Benefits Scheme claims in the Western Australian Data Linkage System) | Record linkage (death registrations in the Western Australian Data Linkage System) | 16.5 |
|  |  |  |  |  |  |  |  |
| Williams  (2011) | NA | Record linkage (the Diabetes Epidemiology Cohort) | ICD-9-CM | 296.2, 296.3, 311, 300.4, 309.0, 293.83, 296.9, 309.1, 296.99, or 301.12 | Record linkage (the Diabetes Epidemiology Cohort) | Record linkage (the Diabetes Epidemiology Cohort) | 4 |
|  |  |  |  |  |  |  |  |
| Richardson  (2008) | NA | Record linkage (Veterans Affairs facility in the southeastern US) | ICD-9 | 296.2, 296.3, 296.5, 300.4, 309.4, 311 | Record linkage (Veterans Affairs facility in the southeastern US) | Record linkage (Beneficiary Identification and Record Location files) | 10 |
|  |  |  |  |  |  |  |  |
| Katon  (2008) | NA | Record linkage (data from Green Ribbon Health beneficiaries) | ICD-9 | 296.2, 296.3, 298.0, 300.4, 309.0, 309.1, 309.28, 311 | Record linkage (data from Green Ribbon Health beneficiaries) | Record linkage (Medicare claims and eligibility files) and information from telephone contact with family | 2 |
|  |  |  |  |  |  |  |  |
| Clouse  (2003) | ≥21 | The National Institute of Mental Health Diagnostic Interview Schedule, version 3 | DSM-III | NA | Diagnostic interview based on the National Diabetes Data Group criteria | Historical data, findings on physical examination, and laboratory, electrocardiographic, and additional test results, and telephone contact established with the subject or a family member | 10 |

CI = confidence interval; CCI = Charlson Comorbidity Index; DEP = Depressive disorder; DEP-DM = Depression-diabetes group; DM-only = Diabetes-only group; NA = not available; N/A = not applicable

^a^ the last name of the first author of the publication, in reverse chronological order of the date of publication.

^b^ for studies which did not provide diagnostic codes, only the diagnostic system specified for the depression diagnosis ascertainment were reported in the table.

**Table S5.**

Matching variables and adjustment variables for the risk estimates in the included studies.

| Author ^a^  (year) | Matching variables | Adjustment variables |  |  |  |  |  |  |
| --- | --- | --- | --- | --- | --- | --- | --- | --- |
|  |  | Sociodemographic  characteristics ^b^ |  | Clinical  characteristics ^c^ |  | Health  behaviours ^d^ |  | Receipt of  healthcare ^e^ |
| Ho  (2025) | Age at incident diabetes, gender, calendar-year period of first diagnosis of diabetes, catchment area, Charlson comorbidity index, hypertension, dyslipidemia, alcohol use disorder, substance use disorders | NA |  | NA |  | NA |  | NA |
|  |  |  |  |  |  |  |  |  |
| Li  (2025) | N/A | Age, gender, ethnicity, Townsend deprivation Index |  | Body mass index, family history of cardiovascular disease, family history of diabetes, diabetes duration, use of hypoglycemic agents, use of lipid-lowering medications |  | Smoking status, alcohol intake |  | NA |
|  |  |  |  |  |  |  |  |  |
| Kim  (2024) | Age, gender, year of index date | Age, gender, income level |  | Body mass index, presence of hypertension, presence of dyslipidemia |  | Smoking status, alcohol consumption, physical activity |  | NA |
|  |  |  |  |  |  |  |  |  |
| Scheuer  (2024) | N/A | Age, gender, education, country of origin |  | Diabetes duration, calendar year |  | NA |  | NA |
|  |  |  |  |  |  |  |  |  |
| Yoo  (2023) | N/A | Age, gender, low income |  | Body mass index, hypertension, dyslipidemia, chronic kidney disease, fasting glucose, duration of diabetes mellitus, insulin use, duration of diabetes mellitus ≥5 years, cardiovascular disease |  | Smoking, drinking, and regular exercise |  | Use of more than three types of oral hypoglycemic agents |
|  |  |  |  |  |  |  |  |  |
| Fleetwood  (2023) | N/A | Age at type 2 diabetes diagnosis, gender, calendar year of type 2 diagnosis, deprivation, ethnicity, health board |  | Body mass index, history of cardiovascular disease, comorbidity, hypertension, high cholesterol, log HbA1c |  | Alcohol use disorder, smoking |  | NA |
|  |  |  |  |  |  |  |  |  |
| Bae  (2022) | N/A | Age, gender, low-income level |  | Body mass index, hypertension, dyslipidemia, chronic kidney disease, heart failure, obstructive sleep apnea, thyroid disease, fasting glucose, diabetes duration, insulin use, and oral hypoglycemic agents |  | Smoking status, alcohol consumption status, regular physical activity |  | NA |
|  |  |  |  |  |  |  |  |  |
| Kim  (2022) | N/A | Age, gender, low income |  | Body mass index, hypertension, dyslipidemia, chronic kidney disease, fasting glucose level, duration of diabetes, insulin use, and number of oral hypoglycemic agents |  | Smoking status, alcohol consumption, physical activity |  | NA |
|  |  |  |  |  |  |  |  |  |
| Scheuer  (2022) | N/A | Age, gender, education, migration status |  | Diabetes duration, calendar year, Charlson Comorbidity Index |  | Substance use disorder |  | NA |
|  |  |  |  |  |  |  |  |  |
| Lunghi  (2021) | Age, gender, Elixhauser comorbidity index | Socioeconomic status, area of residence |  | Depression diagnosis, non-persistence with antidiabetic drugs, number of different drugs in the year before the index date; presence of an anxiety disorder diagnosis in the year before the index date; presence of a cognitive impairment diagnosis in the year before the index date, Elixhauser comorbidity index during follow-up |  | NA |  | Treatment regimen of first antidiabetic drug prescription, number of physician visits in the year before the index date |
|  |  |  |  |  |  |  |  |  |
| Jung  (2021) | N/A | Age, gender |  | Body mass index, hypertension, dyslipidemia, chronic kidney disease, fasting blood glucose, use of insulin, prescription of 2 or more antidiabetic agents |  | Current smoking, alcohol, regular exercise |  | NA |
|  |  |  |  |  |  |  |  |  |
| Wu  (2020) | Year of birth, gender, cohort entry year | N/A |  | The duration between the first diagnosis of diabetes and the initiation of pharmacotherapy, hypertension, dyslipidemia, chronic pulmonary disease, angiotensin-converting enzyme inhibitor, angiotensin receptor blocker, beta blockers, calcium channel blockers, lipid-lowering agents, nonsteroidal anti-inflammatory drugs |  | Alcohol- and substance-related disorders |  | NA |
|  |  |  |  |  |  |  |  |  |
| Lee  (2020) | Age, gender, index year (the year of first diabetes diagnostic date), and Charlson Comorbidity Index | Age, gender |  | Charlson Comorbidity Index, duration of antidepressants use |  | NA |  | NA |
|  |  |  |  |  |  |  |  |  |
| Fernández de Alba  (2020) | N/A | Age, gender |  | Number of non-mental comorbidities, the presence of the other types of mental health comorbidities |  | NA |  | NA |
|  |  |  |  |  |  |  |  |  |
| Chiang  (2020) | N/A | Age, gender |  | Body mass index, socioeconomic status, baseline HbA1c, duration of diabetes, use of antidiabetes drugs, use of corticosteroids |  | Smoking status, alcohol consumptions |  | NA |
|  |  |  |  |  |  |  |  |  |
| Jeong  (2017) | N/A | NA |  | NA |  | NA |  | NA |
|  |  |  |  |  |  |  |  |  |
| Novak  (2016) | N/A | Age, gender, race/ethnicity, and marital status |  | Baseline eGFR, comorbidities at baseline (hypertension, CVD, congestive heart failure, cerebrovascular disease, peripheral vascular disease, lung disease, dementia, rheumatic disease, malignancy, HIV/AIDS, and PTSD) and the use of statins or antihypertensive medications, body mass index, and serum albumin level |  | NA |  | NA |
|  |  |  |  |  |  |  |  |  |
| Lynch  (2014) | N/A | Age, gender, race, marital status, area of residence, geographic region |  | Service connection (representing the degree of disability due to illness or injury that was aggravated by or incurred in military service) |  | NA |  | NA |
|  |  |  |  |  |  |  |  |  |
| Ting  (2013) | NA | Age, gender |  | Duration of diabetes, body mass index, systolic and diastolic blood pressure, HbA1c, lipid profile (LDL cholesterol, HDL cholesterol, triglycerides), Ln (ACR+1), eGFR, use of lipid lowering drugs, ACEI or ARB, other anti-hypertensive drugs, oral anti-diabetic drugs and insulin |  | Smoking status |  | NA |
| Winkley  (2012) | NA | Age, gender, marital status, socioeconomic status |  | Mean HbA1c, ulcer severity |  | Smoking status |  | NA |
|  |  |  |  |  |  |  |  |  |
| Mai  (2011) | 5-year age group, gender, calendar year of being a current elector | 5-year age group, gender, indigenous status, level of social disadvantage (categorised by Index of Relative Socio-Economic Disadvantage scores), level of residential remoteness (categorised by metropolitan, rural and remote derived from the Accessibility/ Remoteness Index of Australia), calendar year at the start of follow-up |  | Charlson index, types of diabetes treatment (no glucose lowering medications, oral hypoglycemic agents [OHA] only, insulin with or without OHA), whether diabetes was identified before the start of follow-up |  | NA |  | NA |
|  |  |  |  |  |  |  |  |  |
| Williams  (2011) | N/A | Age, gender, race/ethnicity, marital status, homelessness, and VA eligibility status |  | Insulin use; other medical conditions (chronic obstructive pulmonary disease, cancer, and acquired immune deficiency syndrome); mental health conditions (posttraumatic stress disorder, anxiety, alcohol abuse, drug abuse, and dementia); cardiovascular risk factors (hypertension and hyperlipidemia); microvascular complications (diabetic eye disease, blindness/low vision, nephropathy, and dialysis); macrovascular complications (ischemic heart disease, prior myocardial infarction, angina, prior coronary artery revascularization, congestive heart failure, prior stroke, transient ischemic attack, prior cerebral artery revascularization, other types of atherosclerosis except lower limb, and prior revascularization of other arteries except lower limb) and foot-specific complications (peripheral arterial disease, prior lower limb artery revascularization, peripheral neuropathy, and foot deformity) |  | NA |  | Number of outpatient visits, number of outpatient mental health visits, and number of hospitalizations |
|  |  |  |  |  |  |  |  |  |
| Katon  (2008) | NA | Age, gender, race |  | Charlson comorbidity index, prior amputation, prior cerebrovascular accident/ cardiovascular event |  | NA |  | NA |
|  |  |  |  |  |  |  |  |  |
| Richardson  (2008) | N/A | Age at baseline, race/ethnicity, marital status, employment status |  | Comorbidity (coronary heart disease, hypertension, stroke, and cancer) |  | NA |  | NA |
|  |  |  |  |  |  |  |  |  |
| Clouse  (2003) | N/A | Age |  | Body mass index, duration of diabetes, glycosylated hemoglobin, hypertension, hyperlipidemia |  | Tobacco use |  | NA |

BD = bipolar disorder; CCI = Charlson Comorbidity Index; NA = not available; N/A = not applicable

^a^ the last name of the first author of the publication, in reverse chronological order of the date of publication.

^b^ refers to covariates such as age, gender, race/ ethnicity, education, marital status, and income.

^c^ refers to covariates such as body mass index, waist circumference, baseline medical comorbidities (e.g. hypertension, dyslipidemia, CCI), duration of diabetes, duration of severe mental illness, and baseline medication prescription.

^d^ refers to covariates such as smoking status, alcohol use, level of physical activity, and dietary pattern.

^e^ refers to covariates such as receipt of diabetes care, blood monitoring, and healthcare visits.

**Table S6.**

Mortality outcome of included studies.

| Author ^a^ (year) | Outcome measure | Depression diagnoses | Cumulative incidence of outcome | Effect size | Risk estimates (95% CI) |
| --- | --- | --- | --- | --- | --- |
| Ho  (2024) | All-cause mortality | Depressive disorder | Depression-diabetes group 20.6%  Diabetes-only group 19.2% | HR | 1.06 (1.02-1.10) |
|  |  |  |  |  |  |
| Li  (2025) | All-cause mortality | Depressive disorder | Depression-diabetes group (NA)  Diabetes-only group (NA) | HR | 1.26 (1.09-1.44) |
|  |  |  |  |  |  |
|  | Cardiovascular disease-specific mortality | Depressive disorder | Depression-diabetes group (NA)  Diabetes-only group (NA) | HR | 1.26 (0.93-1.70) |
|  |  |  |  |  |  |
| Kim  (2024) | All-cause mortality | Depressive disorder | Depression-diabetes group 30.9%  Diabetes-only group 24.9% | HR | 1.31 (1.20-1.44) |
|  |  |  |  |  |  |
| Fleetwood  (2023) | All-cause mortality | Depressive disorder | Depression-diabetes group 23.9%  Diabetes-only group 19.4% | HR | 1.28 (1.22-1.35) |
|  |  |  |  |  |  |
|  | Cardiovascular disease-specific mortality (CVD: ischemic heart disease and cerebrovascular disease) | Depressive disorder | Depression-diabetes group 5.4%  Diabetes-only group 4.5% | HR | 1.29 (1.17-1.42) |
|  |  |  |  |  |  |
| Kim  (2022) | All-cause mortality | Depressive disorders | Depression-diabetes group 11.3%  Diabetes-only group 7.8% | HR | 1.24 (1.22-1.27) |
|  |  |  |  |  |  |
|  | Cardiovascular disease-specific mortality (CVD: myocardial infarction and ischemic stroke) | Depressive disorders | Depression-diabetes group 1.9%  Diabetes-only group 1.3% | HR | 1.15 (1.09-1.21) |
|  |  |  |  |  |  |
| Jung  (2021) | All-cause mortality | Persistent depression (two or more episodes of depression) | Depression-diabetes group 14.4%  Diabetes-only group 6.6% | HR | 1.66 (1.60-1.72) |
|  |  |  |  |  |  |
| Lunghi  (2021) | All-cause mortality | Depressive disorder | Depression-diabetes group 5.54%  Diabetes-only group 2.49 % | OR | 1.32 (1.21-1.45) |
|  |  |  |  |  |  |
|  | Diabetes-related mortality | Depressive disorder | Depression-diabetes group (NA)  Diabetes-only group (NA) | OR | 1.14 (0.61-2.14) |
|  |  |  |  |  |  |
|  | Cardiovascular diseases-related mortality | Depressive disorder | Depression-diabetes group (NA)  Diabetes-only group (NA) | OR | 1.19 (0.99-1.42) |
|  |  |  |  |  |  |
|  | Major cardiovascular events mortality | Depressive disorder | Depression-diabetes group (NA)  Diabetes-only group (NA) | OR | 1.13 (0.89-1.45) |
|  |  |  |  |  |  |
| Fernández de Alba (2020) | All-cause mortality | Depressive disorder | NA | OR | 1.14 (1.07-1.22) |
|  |  |  |  |  |  |
| Wu  (2020) | All-cause mortality | Depressive disorder | Depression-diabetes group 12.0%  Diabetes-only group 8.7% | HR | 1.08 (1.04-1.12) |
|  |  |  |  |  |  |
|  | Diabetes-related mortality | Depressive disorder | Depression-diabetes group 1.1%  Diabetes-only group 1.1% | HR | 0.86 (0.77-0.95) |
|  |  |  |  |  |  |
|  | Circulation death | Depressive disorder | Depression-diabetes group 1.4%  Diabetes-only group 1.3% | HR | 0.96 (0.88-1.06) |
|  |  |  |  |  |  |
|  | Unnatural death | Depressive disorder | Depression-diabetes group 1.4%  Diabetes-only group 0.4% | HR | 2.59 (2.30-2.91) |
|  |  |  |  |  |  |
|  | Suicide | Depressive disorder | Depression-diabetes group 0.8%  Diabetes-only group 0.1% | HR | 5.64 (4.70-6.77) |
|  |  |  |  |  |  |
| Chiang  (2020) | All-cause mortality | Depressive disorder | Depression-diabetes group 12.1%  Diabetes-only group 4.1% | HR | 1.41 (1.2-1.66) (UK Biobank) |
|  |  |  |  |  |  |
|  |  |  | Depression-diabetes group 30.4%  Diabetes-only group 11.5% | HR | 1.23 (1.06-1.42) (Taiwan NDCMP) |
|  |  |  |  |  |  |
| Jeong  (2017) | All-cause mortality | Depressive disorder | Depression-diabetes group 31.16%  Diabetes-only group 22.7% | HR | 1.433 (1.407-1.459) |
|  |  |  |  |  |  |
| Novak  (2016) | All-cause mortality | Depressive disorder | Depression-diabetes group 29%  Diabetes-only group 26% | HR | 1.30 (1.28-1.31) |
|  |  |  |  |  |  |
| Lynch  (2014) | All-cause mortality | Depressive disorder | NA | HR | 1.05 (1.03-1.07) |
|  |  |  |  |  |  |
| Ting  (2013) | All-cause mortality | Depressive disorder | Depression-diabetes group 20.3%  Diabetes-only group 10.6% | HR | 1.07 (0.60-1.90) |
|  |  |  |  |  |  |
| Winkley  (2012) | All-cause mortality | Depressive disorder | NA | HR | 2.09 (1.34-3.25) |
|  |  |  |  |  |  |
|  |  | Minor depression | NA | HR | 1.93 (1.00-3.74) |
|  |  |  |  |  |  |
|  |  | Major depression | NA | HR | 2.18 (1.31-3.65) |
|  |  |  |  |  |  |
| Mai  (2011) | All-cause mortality | Depressive disorder | NA | IRR | 1.54 (1.37-1.73) |
|  |  |  |  |  |  |
|  | Diabetes-related mortality | Depressive disorder | NA | IRR | 1.53 (1.27-1.83) |
|  |  |  |  |  |  |
| Richardson (2008) | All-cause mortality | Depressive disorder | NA | HR | 1.6 (1.3-1.8) |
|  |  |  |  |  |  |
| Katon  (2008) | All-cause mortality | Depressive disorder | NA | HR | 1.36 (1.16-1.59) |

CI = confidence interval; cx = complication; HR = hazard ratio; IRR = incidence rate ratio; OR = odds ratio; NA = not available; N/A = not applicable

**Table S7.**

Complication outcome of included studies.

| Author ^a^ (year) | Outcome | Cumulative incidence of outcome | Effect size | Risk estimates (95% CI) |
| --- | --- | --- | --- | --- |
| Ho  (2024) | Macrovascular complications | Depression-diabetes group 25.4%  Diabetes-only group 25.5% | HR | 1.00 (0.96-1.03) |
|  |  |  |  |  |
|  | Microvascular complications | Depression-diabetes group 10.8%  Diabetes-only group 11.4% | HR | 0.96 (0.90-1.01) |
|  |  |  |  |  |
|  | Cardiovascular complications | Depression-diabetes group 17.1%  Diabetes-only group 17.4% | HR | 0.98 (0.94-1.03) |
|  |  |  |  |  |
|  | Cerebrovascular complications | Depression-diabetes group 11.0%  Diabetes-only group 10.7% | HR | 1.03 (0.97-1.09) |
|  |  |  |  |  |
|  | Peripheral vascular complications | Depression-diabetes group 1.1%  Diabetes-only group 1.5% | HR | 0.72 (0.60-0.85) |
|  |  |  |  |  |
|  | Retinopathy | Depression-diabetes group 2.1%  Diabetes-only group 2.7% | HR | 0.81 (0.71-0.92) |
|  |  |  |  |  |
|  | Nephropathy | Depression-diabetes group 7.3%  Diabetes-only group 8.3% | HR | 0.88 (0.82-0.94) |
|  |  |  |  |  |
|  | Neuropathy | Depression-diabetes group 2.5%  Diabetes-only group 1.8% | HR | 1.44 (1.27-1.62) |
|  |  |  |  |  |
|  | Metabolic complications | Depression-diabetes group 1.1%  Diabetes-only group 0.9% | HR | 1.30 (1.09-1.56) |
|  |  |  |  |  |
| Li  (2025) | Macrovascular complications (composite of outcomes of coronary artery disease, ischemic stroke, and peripheral artery disease) | Depression-diabetes group (NA)  Diabetes-only group (NA) | HR | 1.17 (1.03-1.32) |
|  |  |  |  |  |
|  | Microvascular complications (composite of diabetic kidney disease, diabetic retinopathy, and diabetic neuropathy) | Depression-diabetes group (NA)  Diabetes-only group (NA) | HR | 1.29 (1.14-1.46) |
|  |  |  |  |  |
|  | Cardiovascular complications | Depression-diabetes group (NA)  Diabetes-only group (NA) | HR | 1.26 (1.10-1.45) |
|  |  |  |  |  |
|  | Cerebrovascular complications | Depression-diabetes group (NA)  Diabetes-only group (NA) | HR | 0.90 (0.66-1.23) |
|  |  |  |  |  |
|  | Peripheral vascular complications | Depression-diabetes group (NA)  Diabetes-only group (NA) | HR | 1.07 (0.82-1.40) |
|  |  |  |  |  |
|  | Retinopathy | Depression-diabetes group (NA)  Diabetes-only group (NA) | HR | 0.99 (0.83-1.20) |
|  |  |  |  |  |
|  | Nephropathy | Depression-diabetes group (NA)  Diabetes-only group (NA) | HR | 1.31 (1.09-1.59) |
|  |  |  |  |  |
|  | Neuropathy | Depression-diabetes group (NA)  Diabetes-only group (NA) | HR | 1.96 (1.57-2.45) |
|  |  |  |  |  |
| Kim  (2024) | Composite CV outcomes (first hospital admission for myocardial infarction, ischemic stroke, or CVD-related mortality) | Depression-diabetes group 37.9%  Diabetes-only group 32.8% | HR | 1.23 (1.14-1.33) |
|  |  |  |  |  |
| Scheuer  (2024) | Diabetic ketoacidosis | Depression-diabetes group 0.6%  Diabetes-only group 0.6% | IRR | 1.83 (1.59-2.11) (Type 2 diabetes) |
|  |  |  |  |  |
|  |  | Depression-diabetes group 8%  Diabetes-only group 7.4% | IRR | 1.78 (1.44-2.19) (Type 1 diabetes) |
|  |  |  |  |  |
|  | Hypoglycemia | Depression-diabetes group 2.6%  Diabetes-only group 3.3% | IRR | 1.64 (1.53-1.75) (Type 2 diabetes) |
|  |  |  |  |  |
|  |  | Depression-diabetes group 15.8%  Diabetes-only group 14.6% | IRR | 1.83 (1.58-2.12) (Type 1 diabetes) |
|  |  |  |  |  |
| Yoo  (2023) | Heart failure | Depression-diabetes group 10.3%  Diabetes-only group 5.8% | HR | 1.32 (1.30-1.34) |
|  |  |  |  |  |
| Fleetwood  (2023) | Major cardiovascular event  (ischemic heart disease and cerebrovascular disease) | Depression-diabetes group 9.7%  Diabetes-only group 8.7% | HR | 1.23 (1.13-1.32) |
|  |  |  |  |  |
| Scheuer  (2022) | Nephropathy | Depression-diabetes group 18.7%  Diabetes-only group 22.9% | IRR | 1.07 (1.03-1.10) |
|  |  |  |  |  |
|  | Retinopathy | Depression-diabetes group 2.7%  Diabetes-only group 4.8% | IRR | 0.80 (0.74-0.88) |
|  |  |  |  |  |
|  | Amputation | Depression-diabetes group 1.4%  Diabetes-only group 1.9% | IRR | 1.01 (0.89-1.14) |
|  |  |  |  |  |
|  | Cardiovascular disease | Depression-diabetes group (men) 7.4%  Depression-diabetes group (women) 11.7 %  Diabetes-only group 14.1% | IRR | Men: 1.12 (1.06-1.18)  Women: 1.14 (1.09-1.19) |
|  |  |  |  |  |
| Bae  (2022) | Atrial fibrillation | Depression-diabetes group 4.4%  Diabetes-only group 3.0% | HR | 1.15 (1.12-1.17) |
|  |  |  |  |  |
| Kim  (2022) | Myocardial infarction | Depression-diabetes group 3.4%  Diabetes-only group 2.5% | HR | 1.18 (1.14-1.23) |
|  |  |  |  |  |
|  | Ischemic stroke | Depression-diabetes group 5.0%  Diabetes-only group 3.6% | HR | 1.19 (1.16-1.23) |
|  |  |  |  |  |
| Jung  (2021) | Stroke | Depression-diabetes group  Diabetes-only group | HR | 1.54 (1.46-1.63) |
|  |  |  |  |  |
|  | Myocardial infarction | Depression-diabetes group  Diabetes-only group | HR | 1.38 (1.29-1.49) |
|  |  |  |  |  |
| Wu  (2020) | Microvascular complications | Depression-diabetes group 21.4%  Diabetes-only group 21.2% | HR | 0.99 (0.97-1.02) |
|  |  |  |  |  |
|  | Macrovascular complications | Depression-diabetes group 30.5%  Diabetes-only group 24.3% | HR | 1.35 (1.32-1.37) |
|  |  |  |  |  |
| Lee  (2020) | Hyperglycemic crisis (diabetic ketoacidosis or  hyperosmolar hyperglycemic state) | Depression-diabetes group 1.8%  Diabetes-only group 1.6% | HR | 1.78 (1.56-2.03) |
|  |  |  |  |  |
| Novak  (2016) | Nephropathy (chronic kidney disease) | Depression-diabetes group 21%  Diabetes-only group 18% | HR | 1.18 (1.17-1.20) |
|  |  |  |  |  |
|  | Cerebrovascular disease (ischemic stroke) | Depression-diabetes group 6.4%  Diabetes-only group 4.5% | HR | 1.32 (1.28-1.36) |
|  |  |  |  |  |
|  | Cardiovascular disease (acute myocardial infarction, coronary artery bypass grafting) | Depression-diabetes group 10.3%  Diabetes-only group 6.9% | HR | 1.22 (1.19-1.24) |
|  |  |  |  |  |
| Ting  (2013) | Cardiovascular disease outcomes (coronary heart disease, stroke, peripheral vascular disease) | Depression-diabetes group 20.3%  Diabetes-only group 10.4% | HR | 2.18 (1.45-3.27) |
|  |  |  |  |  |
|  | Stroke | Depression-diabetes group 12.4%  Diabetes-only group 4.8% | HR | 3.55 (2.15-5.84) |
|  |  |  |  |  |
| Williams  (2011) | Any lower limb amputations | Depression-diabetes group 0.8%  Diabetes-only group 0.7% | HR | 1.12 (1.02-1.22) |
|  |  |  |  |  |
| Clouse  (2003) | Coronary heart disease | Depression-diabetes group 38.5%  Diabetes-only group 10.0% | HR | 6.67 (1.26-35.23) |

CI = confidence interval; NA = not available; HR = hazard ratio; IRR = incidence rate ratio; OR = odds ratio

**Table S8.**

Newcastle-Ottawa Scale for assessing the quality of included studies for mortality outcomes in cohort studies and case-control studies.

| Cohort studies  Author  (year) | Selection | | | |  | Comparability | |  | Outcome | | | NOS  score ^a^ | Quality  rating |
| --- | --- | --- | --- | --- | --- | --- | --- | --- | --- | --- | --- | --- | --- |
|  | Representativeness of the exposed cohort | Selection of the non-exposed cohort | Ascertain-ment of exposure | Demonstration that outcome of interest was not present at start of study |  | Controlled for age, gender, and clinical characteristics | Controlled for health behaviour or receipt of healthcare |  | Assessment of outcome | Was follow-up long enough for the outcome to occur? | Adequacy of follow up of cohorts |  |  |
| Ho (2025) | 1 | 1 | 1 | 1 |  | 1 | 1 |  | 1 | 1 | 1 | 9 | Good |
| Li (2025) | 1 | 1 | 1 | 1 |  | 1 | 1 |  | 1 | 1 | 1 | 9 | Good |
| Kim (2024) | 1 | 1 | 1 | 1 |  | 1 | 1 |  | 1 | 1 | 1 | 9 | Good |
| Fleetwood (2023) | 1 | 1 | 1 | 1 |  | 1 | 1 |  | 1 | 1 | 1 | 9 | Good |
| Kim (2022) | 1 | 1 | 1 | 1 |  | 1 | 1 |  | 1 | 1 | 1 | 9 | Good |
| Jung (2021) | 1 | 1 | 1 | 1 |  | 1 | 1 |  | 1 | 1 | 1 | 9 | Good |
| Wu (2020) | 1 | 1 | 1 | 1 |  | 1 | 1 |  | 1 | 1 | 1 | 9 | Good |
| Fernández de Alba (2020) | 1 | 1 | 1 | 1 |  | 1 | 0 |  | 1 | 1 | 1 | 8 | Fair |
| Chiang (2020) (UK) | 1 | 1 | 1 | 1 |  | 1 | 1 |  | 1 | 1 | 1 | 9 | Good |
| Chiang (2020) (Taiwan) | 1 | 1 | 1 | 1 |  | 1 | 1 |  | 1 | 1 | 1 | 9 | Good |
| Jeong (2017) | 1 | 1 | 1 | 1 |  | 0 | 0 |  | 1 | 1 | 1 | 7 | Poor |
| Novak (2016) | 1 | 1 | 1 | 1 |  | 1 | 0 |  | 1 | 1 | 1 | 8 | Good |
| Lynch (2014) | 0 | 1 | 1 | 1 |  | 1 | 0 |  | 1 | 1 | 1 | 7 | Fair |
| Ting (2013) | 0 | 1 | 1 | 1 |  | 1 | 1 |  | 1 | 1 | 1 | 8 | Good |
| Winkley (2012) | 0 | 1 | 1 | 1 |  | 1 | 0 |  | 1 | 1 | 1 | 7 | Fair |
| Mai (2011) | 0 | 1 | 1 | 1 |  | 1 | 0 |  | 1 | 1 | 1 | 7 | Fair |
| Katon (2008) | 0 | 1 | 1 | 1 |  | 1 | 0 |  | 1 | 1 | 1 | 7 | Fair |
| Richardson (2008) | 0 | 1 | 1 | 1 |  | 1 | 0 |  | 1 | 1 | 0 | 7 | Fair |
| Case-control studies | Selection | | | |  | Comparability | |  | Exposure | | |  |  |
| Author  (year) | Adequacy of case definition | Representativeness of the cases | Selection of control from the same population | Definition of control |  | Controlled for age, gender, and clinical characteristics | Controlled for health behaviour or receipt of healthcare |  | Assessment of exposure | Same method of ascertainment for cases and controls | Non-response rate |  |  |
| Lunghi (2021) | 1 | 1 | 1 | 1 |  | 1 | 1 |  | 1 | 1 | 1 | 9 | Good |

^a^ A summary and scoring of the Newcastle-Ottawa Scale is shown Table S7 and Table S8 of the appendices. The quality is categorized into ‘good’, ‘fair’ or ‘poor’ based on the total points as follows: ‘good’ quality required ≥3 points in selection, 2 points in comparability and ≥2 points in outcome; ‘fair’ quality required 2 points in selection, 1 point in comparability and ≥2 points in outcome; ‘poor’ quality reflected ≤1 point in selection or ≤1 point in outcome.

**Table S9.**

Newcastle-Ottawa Scale for assessing the quality of included studies for complication outcomes in cohort studies and case-control studies.

| Cohort studies  Author  (year) | Selection | | | |  | Comparability | |  | Outcome | | | NOS  score ^a^ | Quality rating |
| --- | --- | --- | --- | --- | --- | --- | --- | --- | --- | --- | --- | --- | --- |
|  | Representativeness of the exposed cohort | Selection of the non-exposed cohort | Ascertainment of exposure | Demonstration that outcome of interest was not present at start of study |  | Controlled for age, gender, and clinical characteristics | Controlled for health behaviour or receipt of healthcare |  | Assessment of outcome | Was follow-up long enough for the outcome to occur? | Adequacy of follow up of cohorts |  |  |
| Ho (2025) | 1 | 1 | 1 | 0 |  | 1 | 1 |  | 1 | 1 | 1 | 8 | Good |
| Li (2025) | 1 | 1 | 1 | 1 |  | 1 | 1 |  | 1 | 1 | 1 | 9 | Good |
| Kim (2024) | 1 | 1 | 1 | 1 |  | 1 | 1 |  | 1 | 1 | 1 | 9 | Good |
| Scheuer (2024) | 1 | 1 | 1 | 0 |  | 1 | 0 |  | 1 | 1 | 1 | 7 | Good |
| Yoo (2023) | 1 | 1 | 1 | 1 |  | 1 | 1 |  | 1 | 1 | 1 | 9 | Good |
| Fleetwood (2023) | 1 | 1 | 1 | 1 |  | 1 | 1 |  | 1 | 1 | 1 | 9 | Good |
| Bae (2022) | 1 | 1 | 1 | 1 |  | 1 | 1 |  | 1 | 1 | 1 | 9 | Good |
| Kim (2022) | 1 | 1 | 1 | 0 |  | 1 | 1 |  | 1 | 1 | 1 | 8 | Good |
| Scheuer (2022) | 1 | 1 | 1 | 1 |  | 1 | 0 |  | 1 | 1 | 1 | 8 | Good |
| Jung (2021) | 1 | 1 | 1 | 1 |  | 1 | 1 |  | 1 | 1 | 1 | 9 | Good |
| Wu (2020) | 1 | 1 | 1 | 1 |  | 1 | 1 |  | 1 | 1 | 1 | 9 | Good |
| Lee (2020) | 1 | 1 | 1 | 1 |  | 1 | 0 |  | 1 | 1 | 1 | 8 | Good |
| Novak (2016) | 1 | 1 | 1 | 1 |  | 1 | 0 |  | 1 | 1 | 1 | 8 | Good |
| Ting (2013) | 0 | 1 | 1 | 1 |  | 1 | 1 |  | 1 | 1 | 1 | 8 | Good |
| Williams (2011) | 0 | 1 | 1 | 1 |  | 1 | 0 |  | 1 | 1 | 1 | 7 | Good |
| Clouse (2003) | 0 | 1 | 1 | 0 |  | 1 | 1 |  | 1 | 1 | 1 | 7 | Fair |

^a^ A summary and scoring of the Newcastle-Ottawa Scale is shown Table S7 and Table S8 of the appendices. The quality is categorized into ‘good’, ‘fair’ or ‘poor’ based on the total points as follows: ‘good’ quality required ≥3 points in selection, 2 points in comparability and ≥2 points in outcome; ‘fair’ quality required 2 points in selection, 1 point in comparability and ≥2 points in outcome; ‘poor’ quality reflected ≤1 point in selection or ≤1 point in outcome.

**Figure S1.** Funnel plot for publication bias in meta-analysis of risk of all-cause mortality in diabetes patients with versus without depression.

**Figure S2.** Forest plot for risk of all-cause mortality in diabetes patients with vs without depression stratified by geographic regions.

Studies were arranged in reverse chronological order (first author and their colleagues, year of publication).

CI = confidence interval; RE model = random-effects model; RR = relative risk.

**Figure S3.** Forest plot for risk of all-cause mortality in diabetes patients with vs without depression stratified by study period.

Studies were arranged in reverse chronological order (first author and their colleagues, year of publication).

CI = confidence interval; RE model = random-effects model; RR = relative risk.

**Figure S4.** Forest plot for risk of all-cause mortality in diabetes patients with vs without depression stratified by nature of diabetes sample.

Studies were arranged in reverse chronological order (first author and their colleagues, year of publication).

CI = confidence interval; RE model = random-effects model; RR = relative risk.

**Figure S5.** Funnel plot for publication bias in meta-analysis of risk of any complications in diabetes patients with versus without depression.

**Figure S6.** Forest plot for risk of macrovascular and microvascular complications in diabetes patients with vs without depression.

Studies were arranged in reverse chronological order (first author and their colleagues, year of publication).

CI = confidence interval; RE model = random-effects model; RR = relative risk.

**Figure S7.** Forest plot for risk of specific diabetes complications in diabetes patients with vs without depression.

Studies were arranged in reverse chronological order (first author and their colleagues, year of publication).

CI = confidence interval; RE model = random-effects model; RR = relative risk.

**Figure S8.** Forest plot for risk of any complications in diabetes patients with vs without depression stratified by nature of diabetes.

Studies were arranged in reverse chronological order (first author and their colleagues, year of publication).

CI = confidence interval; RE model = random-effects model; RR = relative risk.

**Figure S9.** Forest plot for risk of any complications in diabetes patients with vs without depression stratified by geographic regions.

Studies were arranged in reverse chronological order (first author and their colleagues, year of publication).

CI = confidence interval; RE model = random-effects model; RR = relative risk.

**Figure S10.** Forest plot for risk of any complications in diabetes patients with vs without depression stratified by study period.

Studies were arranged in reverse chronological order (first author and their colleagues, year of publication).

CI = confidence interval; RE model = random-effects model; RR = relative risk.
